# The MoSR‐MoDde1 Regulatory Axis: A Novel Determinant of DMI Sensitivity and a Target for Sustainable Rice Blast Control

**DOI:** 10.1002/advs.77796

**Published:** 2026-09-16

**Authors:** Fan‐Zhu Meng, Wen‐Kai Wei, Meng‐Yao Wu, Min‐Zheng Cai, Yu‐Fu Wang, Li Zhao, Shuai Meng, Liang‐Fen Yin, Guido Schnabel, Wei‐Xiao Yin, Chao‐Xi Luo

**Affiliations:** ^1^ The Hubei Key Lab of Plant Pathology, College of Plant Science and Technology Huazhong Agricultural University Wuhan China; ^2^ Yazhouwan National Laboratory Sanya China; ^3^ National Joint Local Engineering Laboratory For High‐Efficient Preparation of Biopesticide, College of Forestry and Biotechnology Zhejiang Agriculture and Forestry University Hangzhou China; ^4^ The Experimental Teaching Center of Crop Science, College of Plant Science and Technology Huazhong Agricultural University Wuhan China; ^5^ Department of Plant and Environmental Sciences Clemson University Clemson South Carolina USA

**Keywords:** DMI fungicides, fungicide detoxification, fungicide resistance mechanisms, host‐induced gene silencing (HIGS) rice lines, small‐molecule inhibitors

## Abstract

The transcription factor FgSR functions as a central regulator of ergosterol biosynthesis and 14α‐demethylation inhibitor (DMI) sensitivity in *Fusarium graminearum*, with its orthologous proteins being evolutionarily conserved within Sordariomycetes and Leotiomycetes fungi. In this study, we demonstrate that sterol regulators (SRs) differentially modulate DMI sensitivity across these fungal classes through divergent strategies involving key metabolic genes. In *Magnaporthe oryzae*, MoSR regulates DMI sensitivity through two distinct mechanisms: it directly modulates the transcription of ergosterol biosynthesis genes *MoCYP51A* and *MoCYP51B*, and binds to the DRE (DMI‐responsive element) motif to regulate *MoDde1* (*Magnaporthe oryzae* DMI detoxification enzyme 1), a gene encoding a cytochrome P450 monooxygenase involved in detoxification. Evolutionary analysis indicates that MoDde1 functions as a Sordariomycetes‐specific detoxification factor, capable of enzymatically degrading DMIs. Based on these findings, we developed a small‐molecule D1 targeting MoDde1 and engineered rice lines with reduced *MoDde1* expression using host‐induced gene silencing (HIGS). Both approaches successfully suppressed the enzymatic activity and expression of MoDde1, respectively, demonstrating a promising sustainable strategy for controlling rice blast disease, particularly in conjunction with DMI fungicides.

## Graphical abstract

The transcription factor MoSR governs DMI fungicide sensitivity in *Magnaporthe oryzae* through dual regulation of ergosterol biosynthesis and the detoxification enzyme MoDde1. Targeting MoDde1 via a small‐molecule inhibitor or host‐induced gene silencing enhances DMI efficacy against rice blast, offering a sustainable disease management strategy.

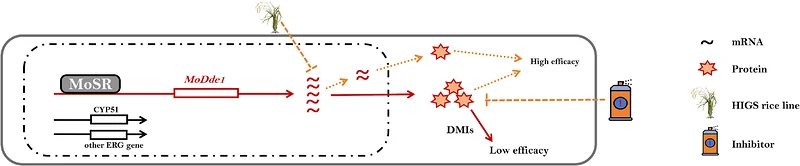

## 1 Introduction

Fungal species engage in dynamic ecological interactions with diverse organisms across the Earth's biosphere, modulating survival strategies through symbiotic, parasitic, and competitive relationships [1]. Notably, pathogenic fungi and their secondary metabolites pose substantial threats to global health and agricultural sustainability, compromising food safety by causing mycotoxin contamination and triggering crop disease epidemics [2]. As essential components of integrated disease management, fungicides play critical roles in protecting crop productivity, and postharvest quality [3]. The 14α‐demethylation inhibitors (DMIs) are a class of fungicides widely used in the control of human, animal, and crop diseases [4]. DMIs inhibit the activity of lanosterol 14α‐demethylase (CYP51 or ERG11), thereby hindering ergosterol biosynthesis and simultaneously increasing ERG3‐catalysed toxic intermediates. This subsequently induces apoptosis and autophagy, ultimately leading to pathogen death [5, 6, 7].

The persistent application of single‐mode‐of‐action fungicides coupled with monoculture agricultural systems accelerates evolution of fungicide resistance in plant pathogens, creating an environment conducive to resistant strain proliferation [8]. DMI resistance mechanisms predominantly involve target‐site (CYP51) alterations, *CYP51* overexpression, or increased efflux pump activity [4]. Specifically, distinct sterol‐sensing regulatory networks control *CYP51* expression across species, e.g., SREBP in *Homo sapiens* [9], Upc2 in *Saccharomyces cerevisiae* [10], and FgSR in *Fusarium graminearum* [11] act as sterol/ergosterol‐sensing transcriptional regulators. Unlike SERBP and UPC2, the FgSR protein does not have a sterol‐sensing structural domain at the C‐terminus, which is dependent on the MAPK cascade signaling pathway to sense changes in ergosterols [11, 12, 13]. FgSR directly binds to the CGAA‐containing cis‐element promoters of the three homologous *CYP51* genes *FgCYP51A*, *FgCYP51B*, and *FgCYP51C*. The FgSR orthologs are exclusively found in fungi belonging to the Sordariomycetes and Leotiomycetes classes. In *Botrytis cinerea*, deletion of the *BcSR* results in a slight increase in the sensitivity to DMIs but is not essential for fungal survival or function [11]. Interestingly, Eurotiomycetes, which are phylogenetically closer to Leotiomycetes, completely lack FgSR orthologous proteins. Instead, they rely on SREBP‐related SrbA and efflux‐linked AtrR transcription factors to adapt to DMIs [14, 15]. The mechanisms by which SR transcription factors regulate diverse DMI responses in the Sordariomycetes and Leotiomycetes lineages are still not well understood.

*Magnaporthe oryzae*, the causal agent of rice blast disease, is one of the most devastating plant pathogens globally, compromising staple crop production and food security [16]. Although DMIs are still the primary fungicides for managing rice blast disease, the emergence of DMI‐resistant *M. oryzae* populations threatens the sustainability of current disease control strategies [17]. Through systematic dissection of the MoSR regulatory network, we identified the cytochrome P450 monooxygenase MoDde1 as a novel detoxification factor that directly modulates sensitivity to DMIs. Mechanistic investigations elucidated that MoDde1 functions as the central effector by which MoSR orchestrates fungal adaptation to DMIs, thereby establishing its crucial role in the evolution of DMI resistance.

## 2 Results

### 2.1 SR Proteins Exert Stronger Regulatory Effects on DMI Sensitivity in Sordariomycetes Compared to Leotiomycetes

**FIGURE 1:**
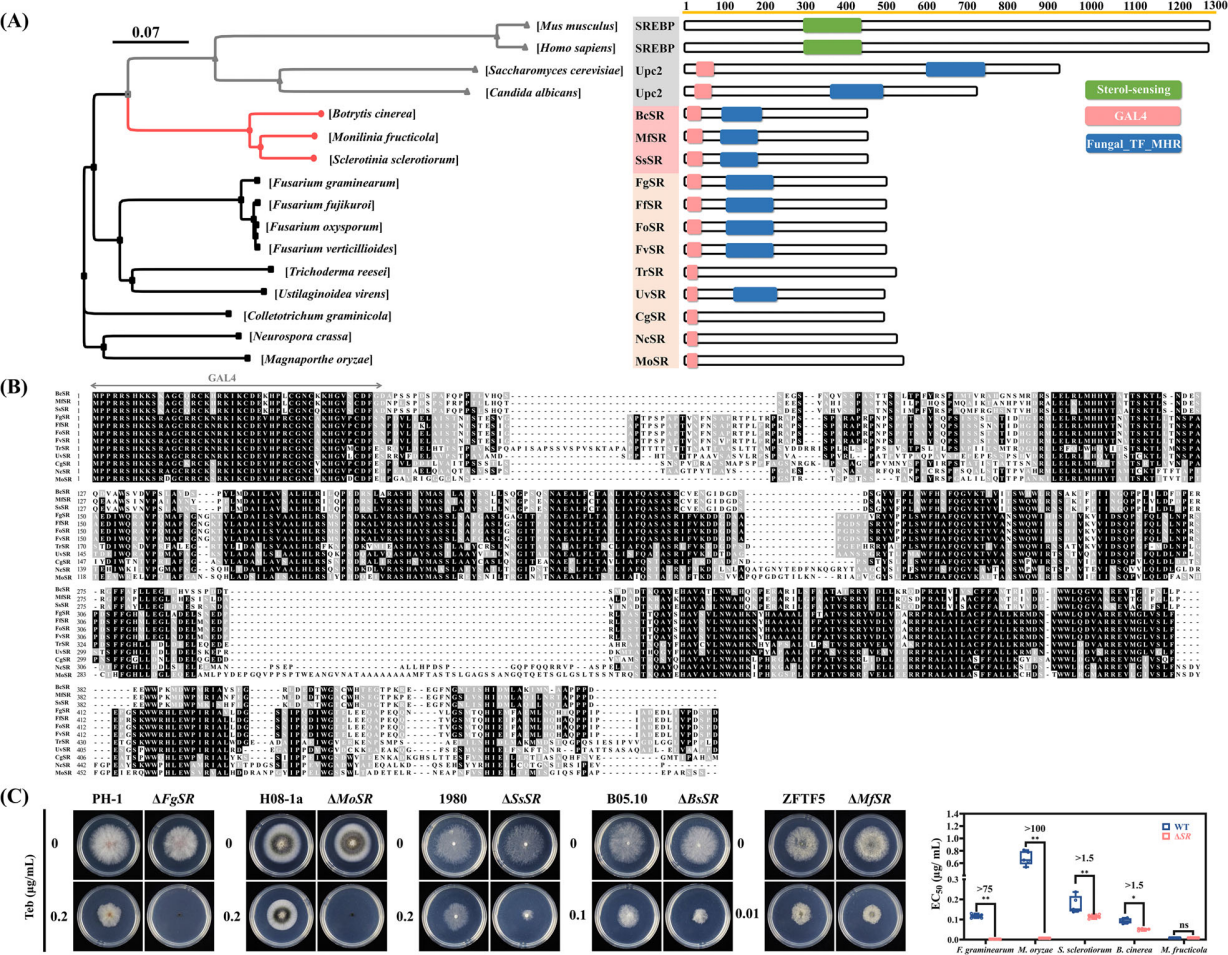
Evolutionary conservation and functional divergence of SR‐mediated DMI sensitivity regulation. (A) Phylogenetic analysis of SR orthologs in ascomycetes (maximum‐likelihood tree, bootstrap = 1000), with structural annotation of conserved GAL4‐like DNA‐binding domains (pink) and fungal‐specific regulatory regions (blue). (B) Multiple sequence alignment of SR proteins from different species. The sequences from top to bottom are listed as follows: *Botrytis cinerea* BcSR, *Monilinia fructicola* MfSR, *Sclerotinia sclerotiorum* SsSR, *Fusarium graminearum* FgSR, *Fusarium fujikuroi* FfSR, *Fusarium oxysporum* FoSR, *Fusarium verticillioides* FvSR, *Trichoderma reesei* TrSR, *Ustilaginoidea virens* UvSR, *Colletotrichum graminicola* CgSR, *Neurospora crassa* NcSR and *Magnaporthe oryzae* MoSR. (C) Differential DMI sensitivity phenotypes in *SR* knockout transformants. A 3‐mm mycelial plug of each strain was inoculated on PDA or PDA amended with different concentrations of Teb and then incubated at 25°C for 2 to 6 days. The left panels show colony morphology, and the right panel shows the statistical results. Data presented are the mean ± SD (*n* = 5). The values in the figures represent the fold difference in EC_50_ between the wild‐type and the corresponding Δ*SR* mutant strains. The symbol “>” indicates that the fold value is higher than the listed number. Statistical significance was analyzed using the t‐test. One asterisk (*) indicates *p* < 0.05, two asterisks (**) indicate *p* < 0.01, and “ns” denotes no significant difference.

Homology searches using FgSR from *F. graminearum* as query identified conserved orthologs across *Fusarium* spp. (*F. graminearum*, *F. fujikuroi*, *F. oxysporum*, *F. verticillioides*), *Trichoderma reesei*, *Ustilaginoidea virens*, *M. oryzae*, *Neurospora crassa*, *Candida graminicola*, and *Sclerotiniaceae* (*B. cinerea*, *Monilinia fructicola*, *Sclerotinia sclerotiorum*) through NCBI BLASTp analysis. Phylogenetic analysis revealed evolutionary conservation of SR proteins within filamentous ascomycetes, forming a clade distinct from sterol‐regulatory transcription factors Upc2/SREBP in Saccharomycetes (*Saccharomyces cerevisiae*, *Candida albicans*) and Metazoa (*Mus musculus*, *Homo sapiens*) (Figure 1A). Structural alignment demonstrated more than 85% sequence identity in the N‐terminal GAL4‐like DNA‐binding domains among orthologs, implying a conserved cis‐element recognition mechanism (Figure 1A,B).

To assess SR‐mediated differential regulation of DMI sensitivity, we generated *SR* knockout transformants in representative Sordariomycetes (*F. graminearum* Δ*FgSR*, *M. oryzae* Δ*MoSR*) and Leotiomycetes (*S. sclerotiorum* Δ*SsSR*, *B. cinerea* Δ*BcSR*, *M. fructicola* Δ*MfSR*). Strikingly, Δ*FgSR* and Δ*MoSR* exhibited more than 50‐fold reductions of EC_50_ values to Teb compared to wild‐type strains (*F. graminearum*, 0.1169 to 0.0016 µg mL^−1^; *M. oryzae*, 0.6803 to 0.0067 µg mL^−1^). In contrast, Leotiomycetes transformants showed minimal sensitivity shifts (*S. sclerotiorum*, 0.1647 to 0.1048 µg mL^−1^; *B. cinerea*, 0.0933 to 0.0506 µg mL^−1^; *M. fructicola*, 0.080 to 0.082 µg mL^−1^) (Figure 1C). These findings revealed the SR‐mediated differential modulation of DMI responses between phylogenetically divergent ascomycete lineages.

### 2.2 Conserved SR‐*CYP51* Regulatory Architecture Is Crucial in Controlling DMI Response in Sordariomycetes and Leotiomycetes

**FIGURE 2:**
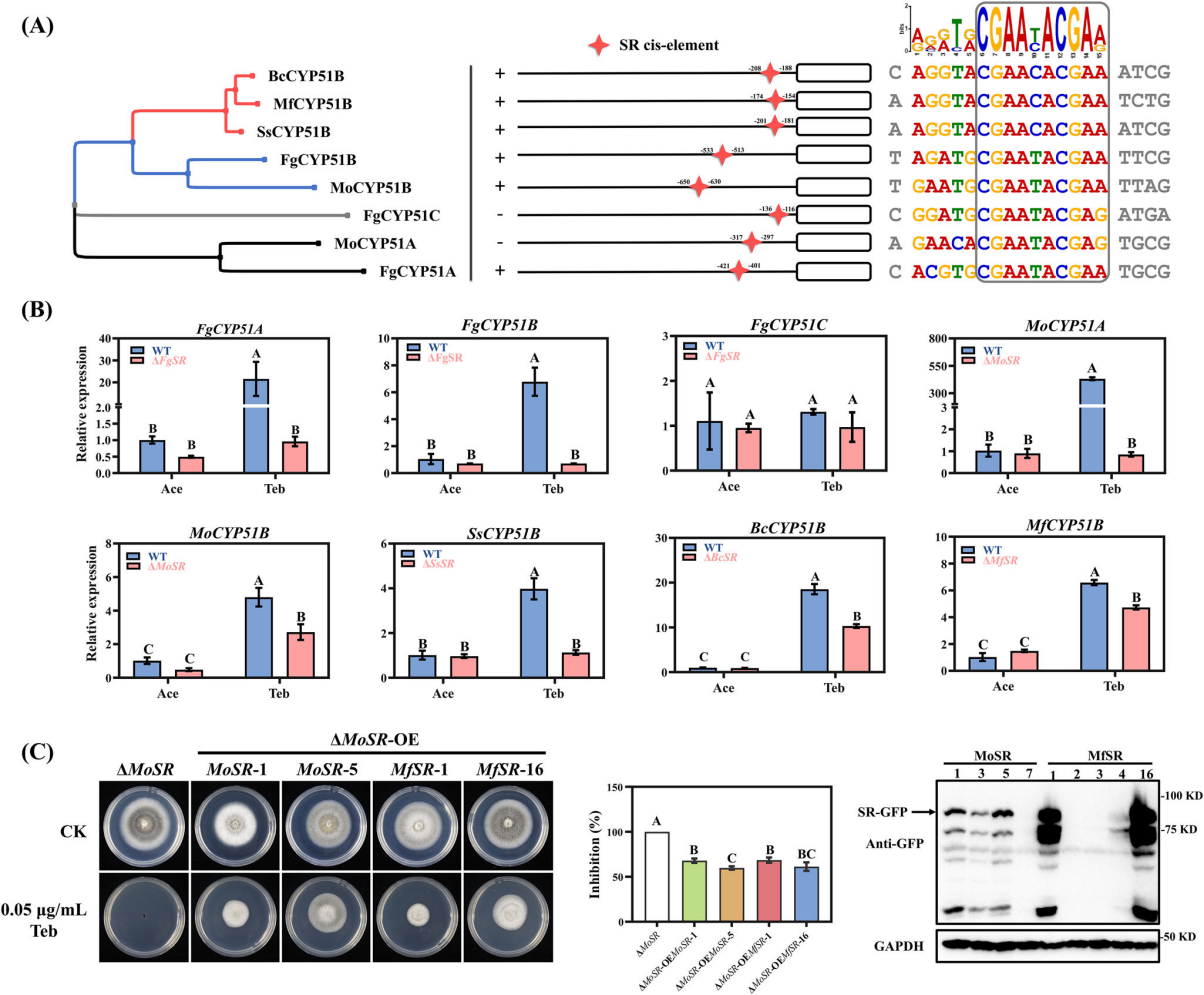
Conserved and divergent roles of SR transcription factors in regulating CYP51 expression and DMI sensitivity. (A) Comparative analysis of *CYP51*s, the location and sequences of SR‐binding cis‐elements in *CYP51* promoters across *F. graminearum*, *M. oryzae*, *S. sclerotiorum*, *B. cinerea*, and *M. fructicola*. All promoters harbor the conserved CGAAT/CACGAA/G motif. The (+) strand denotes the sense orientation, whereas the (−) strand indicates the antisense orientation. Quadrilateral star and box indicate the SR cis‐elements CGAAT/CACGAA/G motif. (B) RT‐qPCR analysis of SR‐regulated *CYP51* expression under acetone (Ace, solvent control) or Teb treatment. (C) Heterologous complementation assay demonstrating MfSR‐mediated regulation of DMI sensitivity in *M. oryzae*. Western blot (GAPDH as loading control) confirms protein expression, while RT‐qPCR data (normalized to *MoActin*) show target gene activation. Data are the mean ± SD (*n* = 3). Bars followed by the same letter are not significantly different according to an LSD test at *p* = 0.01.

DMIs target the 14α‐demethylase CYP51 and inhibit the demethylation of lanosterol, leading to the disruption of ergosterol biosynthesis [18]. Comparative genomic analyses reveal variable *CYP51* paralog architectures across phytopathogenic fungi, e.g., lineage‐specific expansion (1‐3 paralogs) with *CYP51B* maintaining phylogenetic conservation, whereas *CYP51A* emerges as the dominant determinant of DMI sensitivity in species harboring multiple paralogs [19]. Consistent with this paradigm, *F. graminearum* and *M. oryzae* possess tripartite and bipartite *CYP51* systems, respectively, contrasting with *S. sclerotiorum*, *B. cinerea*, and *M. fructicola* retaining a singular *CYP51B* locus (Figure 2A). Based on the high amino acid similarity in the DNA‐binding domains of FgSR and its orthologs, we analyzed promoter cis‐elements of *CYP51* genes across these species. All eight *CYP51* promoters harbored a conserved CGAAT/CACGAA/G motif (Figure 2A), indicating an evolutionarily conserved SR‐mediated regulatory mechanism in Sordariomycetes and Leotiomycetes. RT‐qPCR confirmed SR‐dependent activation of *CYP51A* and *CYP51B* upon exposure to the DMI fungicide Teb, except for *FgCYP51C*. Notably, *CYP51B* expression displayed a milder attenuation in Δ*SR* strains compared to *CYP51A*, suggesting that reduced regulatory efficacy toward *CYP51B* may underlie the limited SR‐mediated control of DMI sensitivity in Leotiomycetes fungi. Although Δ*MfSR* strains showed no significant shift in Teb sensitivity, *MfCYP51B* expression decreased by 25% under Teb treatment (Figure 2B). Heterologous expression of *MfSR* in Δ*MoSR *partially restored Teb sensitivity, similar to the Δ*MoSR*‐OE*MoSR* complementation (Figure 2C). This functional rescue implies that SR‐mediated DMI sensitivity is modulated by the expression dynamics and copy number of downstream targets.

### 2.3 MoSR Is a Master Regulator of DMI Sensitivity in *M. oryzae*

**FIGURE 3:**
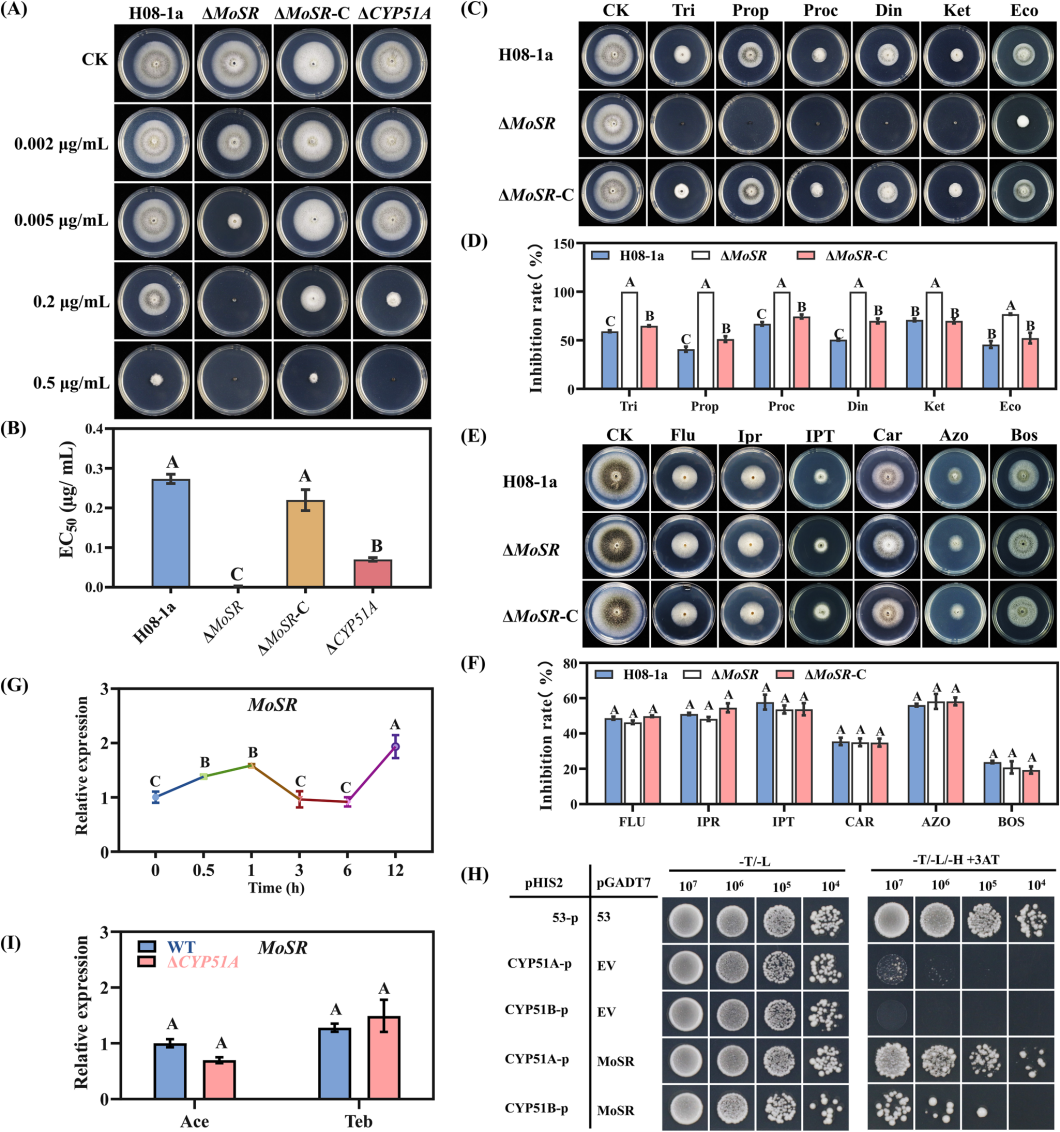
MoSR is a major transcription factor regulating DMI sensitivity in *M. oryzae*. (A) The Δ*MoSR* knockout transformants were more sensitive to Teb compared to the Δ*CYP51* knockout transformants. (B) EC_50_ value comparisons of *MoSR* and *MoCYP51A*‐related transformants to Teb. (C) MoSR regulates the sensitivity to other DMI fungicides in *M. oryzae*. (D) Statistical analysis of sensitivity to other DMIs in *MoSR‐*related transformants. (E) Multi‐fungicide sensitivity testing of *MoSR‐*related transformants. Transformants were inoculated on PDA or PDA amended with 4 µg mL^−1^ fludioxonil (Flu), 20 µg mL^−1^ iprodione (Ipr), 5 µg mL^−1^ Isoprothiolane (IPT), 0.3 µg mL^−1^ carbendazim (Car), 1 µg mL^−1^ Azoxystrobin (Azo) and 30 µg mL^−1^ boscalid (Bos) and then incubated at 27°C for 5 days. (F) Statistical analysis of sensitivity to other fungicides in *MoSR‐*related transformants. (G) RT‐qPCR demonstrates transcriptional stability of *MoSR* under Teb stress. (H) Yeast one‐hybrid assays confirmed the direct binding between MoSR and “CGAATACGAA/G” cis‐elements in *MoCYP51A* and *MoCYP51B* promoters (Positive control: pHIS2‐53 and pGADT7‐53p). (I) *MoCYP51A* deletion did not alter *MoSR* expression under Teb stress. The *MoActin* gene was used as the internal reference for normalization. Data presented are the mean ± SD (*n* = 3). Bars followed by the same letter are not significantly different according to an LSD test at *p* = 0.01.

To demonstrate the evolutionary conservation of SR‐mediated DMI adaptation, we systematically deconstructed the MoSR regulatory network in *M. oryzae*. Gene disruption and complementation strategies (Figure S1A,B) yielded Δ*MoSR* transformants exhibiting 100‐fold hypersensitivity to Teb, while the sensitivity was rescued to the wild‐type level in Δ*MoSR*‐C complemented strains (Figure 3A,B). Strikingly, Δ*MoSR* displayed selective hypersensitivity to DMIs without cross‐sensitivity to fungicides with different modes of action, confirming the specificity of MoSR‐mediated DMI adaptation (Figure 3C–F). The transcription level of *MoSR* remains stable under Teb treatment (Figure 3G). Phenotypic profiling revealed unaffected saprophytic fitness in Δ*MoSR* (unchanged mycelial growth, conidiation, stress tolerance), but impaired pathogenicity‐associated morphogenesis (reduced appressorium formation and virulence) (Figure S2).

A previous study indicated that *MoCYP51A* is a core determinant of DMI sensitivity in *M. oryzae*, while deletion of *MoCYP51B* has little phenotypic effect [20]. Yeast one‐hybrid assays confirmed that the MoSR directly bound to *MoCYP51A*/*B* promoters (Figure 3H). Considering that Δ*MoCYP51A* only exhibited intermediate Teb hypersensitivity with a much higher EC_50_ value than that of Δ*MoSR*, and *MoSR* transcription remained stable under Teb stress in the Δ*MoCYP51A* background (Figure 3I), implying that in addition to *MoCYP51A*, other unknown target genes of MoSR are likely involved in regulating DMI fungicide sensitivity in *M. oryzae*. In other words, MoSR coordinates DMI sensitivity through dual transcriptional controls: it directly activates *MoCYP51A* expression and simultaneously regulates other unknown pathways in *M. oryzae*.

### 2.4 *MoDde1*, A Downstream Target of MoSR, Critically Modulates DMI Sensitivity in *M. oryzae*

**FIGURE 4:**
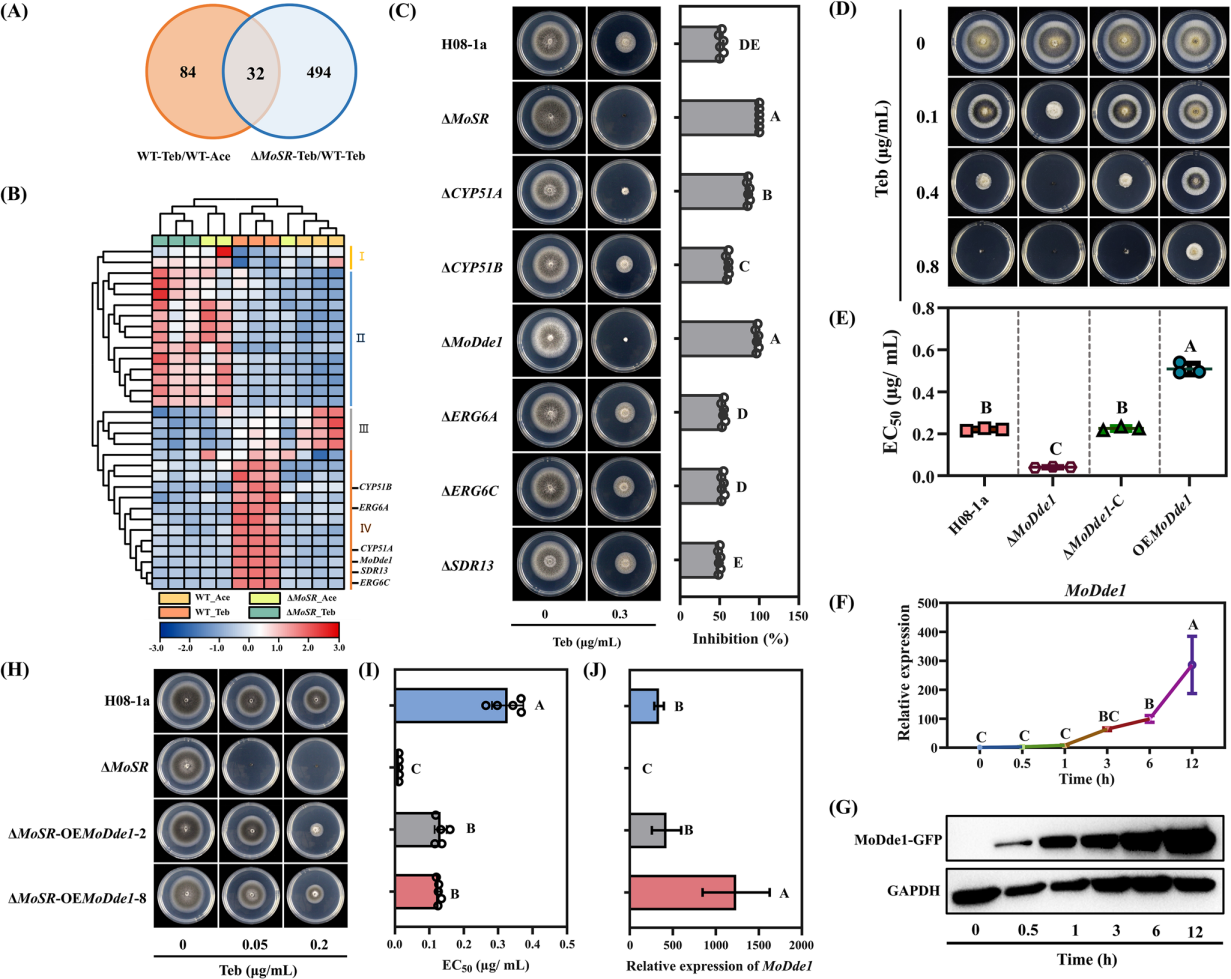
*MoDde1* is a crucial target gene of MoSR governing DMI sensitivity in *M. oryzae*. (A) Venn diagram of MoSR‐dependent differentially expressed genes (DEGs) under Teb treatment. (B) Hierarchical clustering heatmap showing expression patterns of DEGs. (C) Wild‐type H08‐1a and knockout transformants (Δ*MoSR*, Δ*MoCYP51A*, Δ*MoCYP51B*, Δ*MoDde1*, Δ*MoERG6A*, Δ*MoERG6C*, Δ*MoSDR13*) were cultured on PDA plates amended with 0.4 µg mL^−1^ Teb or control PDA. Data presented are the mean ± SD (*n* = 6). (D) Fungicide sensitivity assays of *MoDde1*‐related transformants to Teb (0.1, 0.4, and 0.8 µg mL^−1^). Wild‐type H08‐1a and transformants were cultured on PDA plates with/without fungicides at 27°C for 5 days. (E) Statistical analysis of the EC_50_ value of Teb for MoSR‐related transformants. Data presented are the mean ± SD (*n* = 3). (F) RT‐qPCR analysis of *MoDde1* expression in wild‐type H08‐1a under Teb treatment. (G) Western blot detection of *MoDde1* expression in complemented strain Δ*MoDde1*‐C after Teb treatment. GAPDH served as an internal control. Each strain was cultured in PDB for 48 h and then treated with 0.5 µg mL^−1^ Teb for 0, 0.5, 1, 3, 6, and 12 h, respectively. (H) Fungicide sensitivity assays of Δ*MoSR*‐OE*MoDde1* transformants to Teb. (I) Statistical analysis of the EC_50_ value of Teb for Δ*MoSR*‐OE*MoDde1* transformants. Data presented are the mean ± SD (*n* = 5). (J) RT‐qPCR analysis of *MoDde1* expression in Δ*MoSR*‐OE*MoDde1* transformants. Data were normalized to *MoActin* and presented as mean ± SD (*n* = 3). Bars followed by the same letter are not significantly different according to an LSD test at *p* = 0.01.

To further identify downstream targets of MoSR for modulating DMI sensitivity, wild‐type H08‐1a and Δ*MoSR* knockout transformant were treated with 0.5 µg mL^−1^ Teb or acetone (control) for 4 h. RNA‐Seq analysis revealed 32 differentially expressed genes (DEGs) between Δ*MoSR* and wild‐type under Teb exposure (Figure 4A). Hierarchical clustering grouped 32 DEGs into four expression clusters, with Cluster IV (13 genes, including *MoCYP51A*) exhibiting an upregulation pattern under MoSR‐dependent Teb response (Figure 4B). Six DEGs (*MoCYP51A*, *MoCYP51B*, *MoERG6A*, *MoERG6C*, *MoDde1* (DMI detoxification enzyme in *M. oryzae*), *MoSDR13*) showing high fold‐changes and high basal expression were prioritized for functional validation. These genes encode four core enzymes of the ergosterol synthesis pathway, i.e., CYP51 enzymes MoCYP51A and MoCYP51B, the sterol 24‐C‐methyltransferases MoERG6A and MoERG6C, and two non‐ergosterol synthesis pathway enzymes, i.e., the cytochrome P450 enzyme MoDde1 and the dehydrogenase/reductase SDR13, respectively. Phenotypic screening of knockout transformants (Δ*MoCYP51A*, Δ*MoCYP51B*, Δ*MoDde1*, Δ*MoERG6A*, Δ*MoERG6C*, Δ*MoSDR13*) revealed that Δ*MoDde1* and Δ*MoCYP51A* were significantly sensitive to Teb, whereas the sensitivity of the other transformants showed no significant alterations or only marginal changes (Figure 4C).

To characterize MoDde1 roles, complementation (Δ*MoDde1*‐C) and overexpression (OE*MoDde1*) transformants were generated utilizing the 2.2‐kb natural promoter and the 1.5‐kb *MoH3* gene promoter, respectively (Figure S3). Dose‐response assays demonstrated that Δ*MoDde1* displayed a hypersensitive response to Teb with a 10‐fold reduction in EC_50_ value, while sensitivity restored in Δ*MoDde1*‐C to the wild‐type level and exacerbated resistance in OE*MoDde1* (Figure 4D,E).

Since cytochrome P450s are generally induced by their substrate drugs to facilitate detoxification, we assessed the dynamics of *MoDde1* expression under Teb treatment. In a complemented strain expressing C‐terminal GFP‐tagged MoDde1, both transcript and protein levels increased upon exposure to 0.5 µg mL^−1^ Teb (Figure 4F,G). Cross‐resistance profiling against six other DMIs revealed that Δ*MoDde1* exhibited hypersensitivity to five of them (prochloraz, ketoconazole, propiconazole, econazole, diniconazole), but not triadimefon (Figure S4A,B). Δ*MoDde1* and OE*MoDde1* showed unaltered sensitivity to six non‐DMI fungicides (fludioxonil, iprodione, isoprothiolane, carbendazim, azoxystrobin, boscalid) (Figure S4C,D), indicating that MoDde1 confers specific resistance to DMIs rather than broad multidrug resistance, unlike some P450s (e.g., ShCYP561/65/68).

To clarify the contribution of MoDde1 to the MoSR regulatory pathway, we constructed a *MoDde1*‐overexpressing strain in the Δ*MoSR* background (designated as Δ*MoSR*‐OE*MoDde1*). Results demonstrated that the sensitivity of Δ*MoSR*‐OE*MoDde1* strains to Teb was restored to 40% of that of the wild‐type strain H08‐1a (Figure 4H–J). This finding further confirms that *MoDde1* is a crucial target gene of MoSR determining DMI sensitivity in *M. oryzae*.

Collectively, these results demonstrate that MoDde1 is a key DMI‐specific resistance determinant in *M. oryzae*, likely involved in direct fungicide detoxification rather than acting through ergosterol biosynthesis‐dependent regulatory mechanisms.

### 2.5 *MoDde1* Is a Core Target Gene of MoSR in Regulating DMI Sensitivity and Its DRE Motif Binds Directly to MoSR

**FIGURE 5:**
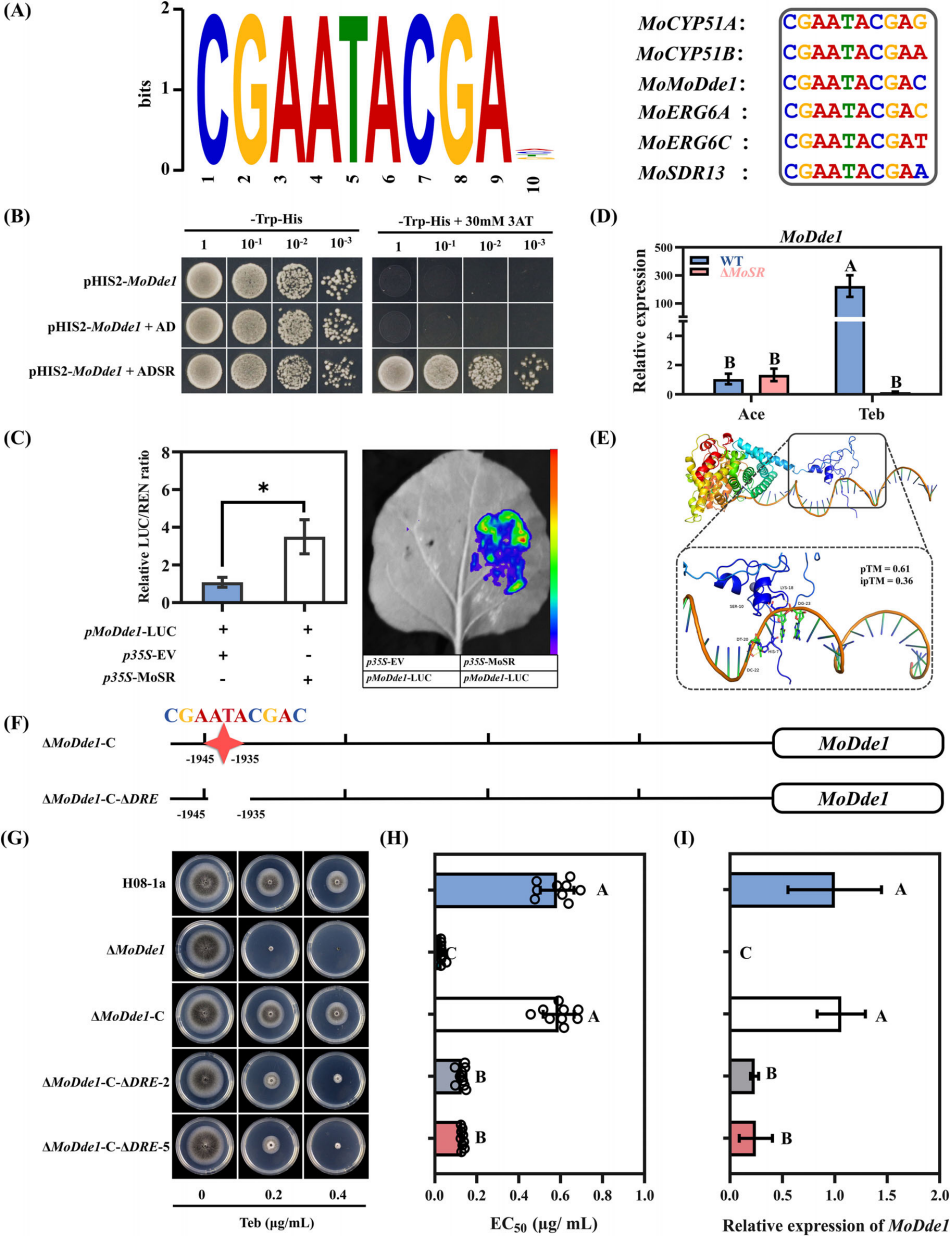
MoSR directly binds the *MoDde1* promoter DRE motif. (A) MEME motif analysis was performed to characterize the conserved DRE motif in the promoter regions of MoSR target genes. (B) A yeast one‐hybrid assay confirmed the binding between MoSR and the *MoDde1* promoter. (C) Dual‐luciferase assay validating MoSR interaction with the *MoDde1* promoter containing a DRE motif. (D) RT‐qPCR was performed to validate the regulatory role of MoSR in *MoDde1* expression under Teb induction. (E) Protein–DNA interaction between MoSR and the DRE motif within the MoDde1 promoter was predicted using Protenix. https://protenix‐server.com. (F) Schematic diagram illustrating the DRE motif deletion strategy in the *MoDde1* promoter region. (G) Analysis of fungicide sensitivity of Δ*MoDde1*‐C‐Δ*DRE* transformants to the DMI fungicide Teb. (H) Statistical analysis of the EC_50_ value of Teb for Δ*MoDde1*‐C‐Δ*DRE* transformants. Data are the mean ± SD (*n* = 9). (I) RT‐qPCR analysis of *MoDde1* expression in Δ*MoDde1*‐C‐Δ*DRE* transformants. Data were normalized to *MoActin* and presented as mean ± SD (*n* = 3). Statistical analyses of Figure 5D, H and I were conducted via the LSD method, where values marked with identical letters show no significant difference, with uppercase letters denoting significance at *p* = 0.01. Statistical analysis for Figure 5C was performed using the t‐test, where one asterisks (*) denote *p* < 0.05.

The drug‐responsive element is a core cis‐acting element indispensable for the transcriptional upregulation of genes in response to exogenous fungicide stress. Specifically, a conserved cis‐acting element containing the consensus sequence “CGAATACGA” is ubiquitously present in the promoter regions of all core target genes downstream of MoSR. Therefore, we hereby designate it as the DRE (DMI‐responsive element) (Figure 5A). To determine whether MoSR directly interacts with the DRE motif in the *MoDde1* promoter, we employed multiple molecular and genetic approaches. The yeast one‐hybrid (Y1H) assay demonstrated that MoSR bound specifically to the promoter fragment containing the DRE motif (Figure 5B). This physical interaction was functionally confirmed using a dual‐luciferase reporter system, where MoSR and *MoDde1* promoter significantly activated the expression of the luciferase (Figure 5C). Consistent with these findings, RT‐qPCR revealed that *MoDde1* expression was positively regulated by MoSR activity, showing upregulation upon MoSR activation and downregulation in its absence under Teb stress (Figure 5D). Molecular docking analysis further confirmed that the GAL4‐like DNA‐binding domain of MoSR is capable of binding to the DRE motif (Figure 5E). To further validate the functional indispensability of the DRE motif, we generated a DRE motif‐deleted mutant strain with the deletion introduced in the *MoDde1* promoter region (designated as Δ*MoDde1*‐C‐Δ*DRE*) (Figure 5F). Phenotypic and expression analyses revealed that DRE motif deletion not only significantly increased the sensitivity to Teb (Figure 5G,H) but also led to a marked suppression of *MoDde1* transcription under Teb induction (Figure 5I). Together, these results demonstrate that MoSR directly binds the DRE element in the *MoDde1* promoter to transcriptionally activate *MoDde1*, thereby modulating DMI fungicide sensitivity.

### 2.6 *MoDde1* Is Not Essential for Environmental Fitness in *M. oryzae*

The evolution of fungicide resistance in pathogenic fungi is frequently associated with fitness trade‐offs, which may limit the proliferation of resistant populations. To investigate the role of *MoDde1* in the ecological adaptability of *M. oryzae*, we evaluated phenotypic traits of *MoDde1* knockout and complemented transformants, including mycelial growth, sporulation, appressorium formation, pathogenicity, and stress tolerance. As illustrated in Figure S5, the knockout transformant Δ*MoDde1*did not show significant differences with the wild‐type strain H08‐1a for growth rate, sporulation, appressorium formation, pathogenicity, or sensitivity to tested environmental stresses except sorbitol. These results demonstrate that *MoDde1* is dispensable for maintaining environmental fitness in *M. oryzae*.

### 2.7 MoDde1 Metabolizes DMI Fungicides, Rather Than Affecting Ergosterol Synthesis

**FIGURE 6:**
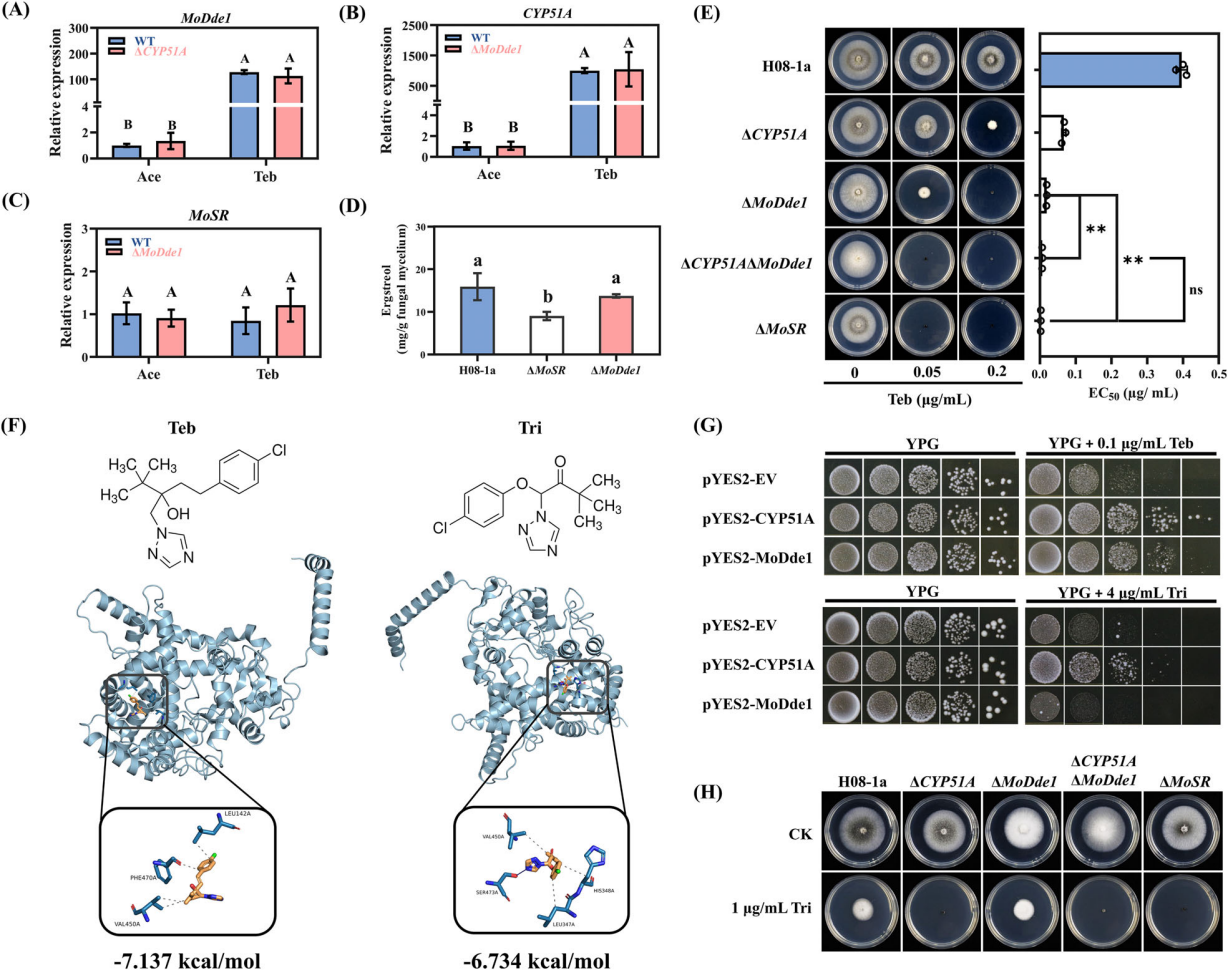
MoDde1 is a detoxification enzyme for DMIs. (A) Expression of *MoDde1* in Δ*MoCYP51A* transformants was detected using RT‐qPCR. (B) Expression of *MoCYP51A* in Δ*MoDde1* transformants was detected using RT‐qPCR. (C) Expression of *MoSR* in Δ*MoDde1* transformants was detected using RT‐qPCR. Data were normalized to *MoActin* and presented as mean ± SD (*n* = 3). (D) The MoDde1 did not participate in ergosterol synthesis. The ergosterol content of each strain was determined with HPLC after 48 h of growth in PDB and 2 h of treatment with 0.4 µg mL^−1^ Teb. Data are the mean ± SD (*n* = 3). (E) Detection of the Teb sensitivity of different types of transformants. A 3‐mm mycelial plug of each strain was inoculated on PDA or PDA amended with 0, 0.05, or 0.2 µg mL^−1^ Teb and then incubated at 27°C for 5 days. Data are the mean ± SD (*n* = 3). (F) MoDde1 binds strongly with Teb rather than Tri based on molecular docking. (G) Heterologous expression of *MoDde1* in *Saccharomyces cerevisiae* BY4741 significantly decreased sensitivity to Teb but not Tri. (H) MoDde1 did not affect the Tri sensitivity in *M. oryzae*. Statistical analyses of Figure 6A–D were conducted via the LSD method, where values marked with identical letters show no significant difference, with uppercase and lowercase letters denoting significance at *p* = 0.01 and *p* = 0.05 respectively. Statistical analysis for Figure 6E was performed using the t‐test, where two asterisks (**) denote *p* < 0.01 and “ns” indicates no significant difference.

FgSR functions as a master transcriptional regulator of ergosterol biosynthesis in *F. graminearum*. Our study confirms that MoSR retains this conserved role, governing the expression of core ergosterol pathway genes, including *MoCYP51A*, *MoCYP51B*, *MoERG6A*, and *MoERG6C*. To determine whether *MoDde1* influences ergosterol biosynthesis, we analyzed the transcription of key pathway genes and quantified ergosterol content in Δ*MoDde1* mutants via RT‐qPCR and HPLC (High‐Performance Liquid Chromatography). RT‐qPCR revealed that the deletion of *MoCYP51A* or *MoDde1* alone had neither impact on the transcriptional levels of each other (Figure 6A,B), nor on that of their upstream regulatory factor MoSR (Figure 3I and Figure 6C). HPLC results indicated that ergosterol levels in Δ*MoDde1* remained comparable to the wild‐type strain H08‐1a, whereas Δ*MoSR* showed a consistent 20% reduction (Figure 6D). Strikingly, genetic epistasis analysis uncovered a strong synergistic interaction between *MoCYP51A* and *MoDde1*. The double knockout Δ*MoCYP51A*Δ*MoDde1* exhibited a 60‐fold decrease in Teb EC_50_, dramatically exceeding the individual effects of single mutants (5‐ and 20‐fold reductions, respectively), comparable to the Δ*MoSR* (Figure 6E). These results demonstrate that MoDde1 modulates DMI sensitivity through a mechanism independent of ergosterol biosynthesis, defining a parallel, non‐canonical resistance pathway downstream of MoSR.

Cytochrome P450 enzymes constitute core catalytic components of xenobiotic metabolic systems, mediating oxidative transformations of both endogenous substrates (fatty acids, sterols) and exogenous substrates or xenobiotics (drugs, phytochemicals, environmental pollutants) through conserved heme‐dependent mechanisms. Given that MoDde1 does not influence ergosterol biosynthesis, we hypothesize that it may directly interact with DMI fungicides to mediate detoxification. Molecular docking analysis revealed strong binding affinity between MoDde1 and Teb, with a calculated binding energy of –7.137 kcal mol^−1^, whereas binding to triadimefon (Tri) was weaker (−6.734 kcal mol^−1^) (Figure 6F). Subsequent DARTS (drug affinity responsive target stability) assays further validated these binding relationships in vitro (Figure S7). Functional validation in *Saccharomyces cerevisiae* BY4741 showed that heterologous expression of *MoDde1* significantly reduced yeast sensitivity to Teb, but not to Tri (Figure 6G). This result aligns with the DMI‐specific resistance profile observed in *MoDde1* mutants of *M. oryzae* (Figure 6H), supporting a model in which MoDde1 confers resistance through direct binding and modification of specific DMI fungicides, with the notable exception of Tri.

### 2.8 MoDde1 Orthologs Display Substantial Evolutionary Conservation across Phylogenetically Affiliated Filamentous Fungal Species

Given that MoDde1 mediates enhanced detoxification capacity against DMIs in *M. oryzae*, the phylogenetic conservation of Dde1 proteins among other ascomycete fungi requires examination. Phylogenetic analysis revealed selective preservation of MoDde1 orthologs in specific Sordariomycetes species (e.g., *Fusarium* spp.), while being conspicuously absent in Leotiomycetes lineages (Figure S6A). The acquisition of *MoDde1* and *MoCYP51A* genes enhanced the capacity of SR homologs to modulate DMI sensitivity in Sordariomycetes. We overexpressed *FgDde1* (*FGSG_11536*), the ortholog of *MoDde1*, in *F. graminearum*, as well as *MoDde1* in Leotiomycetes *B. cinerea*. Results showed that the overexpression of either *FgDde1* or *MoDde1* markedly enhanced the resistance of filamentous fungi to DMIs, except for Tri, confirming the conserved detoxification function of these orthologous proteins (Figure S6B–H).

### 2.9 *FgCYP637A1* Functions in a Similar Role to *MoDde1* in *F. graminearum*

**FIGURE 7:**
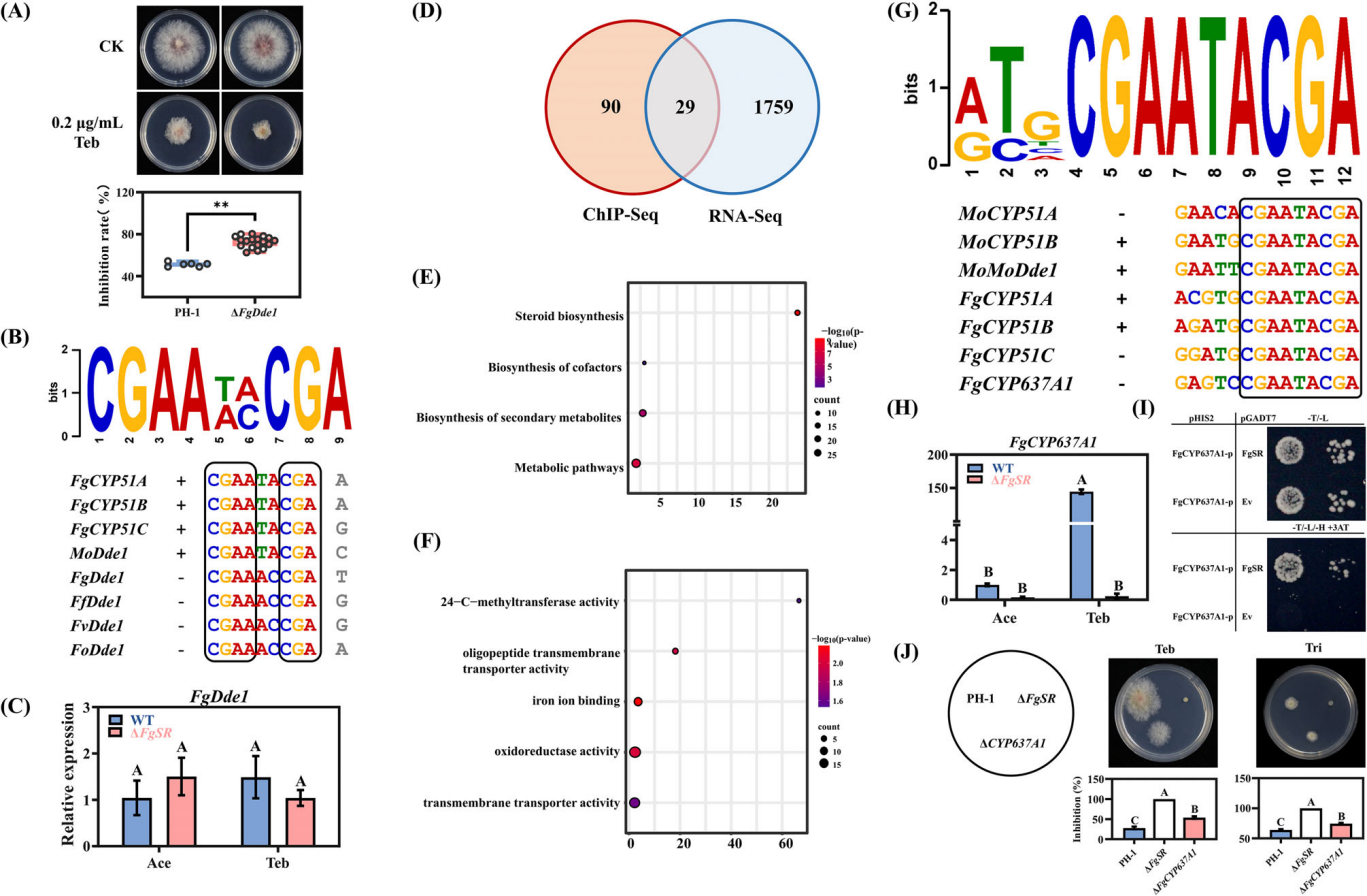
*FgCYP637A1* is one of the key target genes of FgSR regulating DMI sensitivity in *F. graminearum*. (A) Knockout transformants Δ*FgDde1* increased sensitivity to Teb. Data are the mean ± SD (*n* = 6). Δ*FgDde1* indicates three independent deletion mutants (Δ*FgDde1*‑1, Δ*FgDde1*‑2, Δ*FgDde1*‑3). (B) MEME‐based identification of conserved DRE in promoters of *MoDde1* homologs across *Fusarium* species. (C) RT‐qPCR confirmed no significant downregulation of *FgDde1* transcript levels in the Δ*FgSR*. Data were normalized to *FgActin* and presented as mean ± SD (*n* = 3). (D) Integrative analysis of co‐differentially expressed genes (C‐DEGs) identified through combined FgSR‐related RNA‐seq and ChIP‐seq datasets. (E) KEGG enrichment analysis of C‐DEGs. (F) Gene ontology enrichment analysis of C‐DEGs. (G) MEME motif discovery identified putative DRE in the *FgCYP637A1* promoter. (H) *FgCYP637A1* expression is markedly reduced in Δ*FgSR*. Data were normalized to *FgActin* and presented as mean ± SD (*n* = 3). (I) Yeast one‐hybrid confirmed that FgSR binds to the DRE motif of the *FgCYP637A1* promoter. (J) Knockout transformants Δ*FgCYP637A1* exhibited hypersensitivity to Teb (0.2 µg mL^−1^) and Tri (5.0 µg mL^−1^). Data are the mean ± SD (*n* = 4). Statistical analysis for Figure 7A was performed using the t‐test, where two asterisks (**) denote *p* < 0.01. Statistical analyses of Figure 7C, 7H and 7J were conducted via the LSD method; different letters indicate significant differences at *p* = 0.01.

As research continues, we found a divergent regulatory mechanism of DMI resistance between *F. graminearum* and *M. oryzae*. While overexpression of *FgDde1* enhanced DMI resistance in *F. graminearum*, the knockout transformants Δ*FgDde1* only exhibited increased Teb sensitivity to a certain degree, contrasting sharply with the pronounced hypersensitivity observed in the knockout transformants Δ*MoDde1* in *M. oryzae* (Figure 7A). Intriguingly, sequence analysis identified TA→AC mutations within the DRE motif of the *FgDde1* promoter (Figure 7B). Further RT‐qPCR analyses confirmed that *FgDde1* expression remained unresponsive to DMI exposure and was not transcriptionally regulated by FgSR (Figure 7C). These findings demonstrate that the evolutionary conservation of DRE motifs and MoDde1 orthologs is equally essential for the acquisition of novel fungicide‐responsive adaptive functions in phytopathogenic fungi.

In *F. graminearum*, while FgDde1 showed no significant influence on DMI sensitivity, the knockout transformant Δ*FgSR* exhibited extreme hypersensitivity to DMIs, suggesting the existence of other FgSR‐regulated target genes that may perform similar detoxification functions like *MoDde1*. Through integrative analysis of published ChIP‐seq and RNA‐seq datasets under Teb stress, we identified 29 DEGs modulated by FgSR (Figure 7D). Functional annotation revealed these genes are predominantly involved in ergosterol biosynthesis pathways or display transport/oxidase activities (Figure 7E,F). Notably, the cytochrome P450‐encoding gene *FgCYP637A1* contained a conserved “CGAATACGA” DRE in its promoter region (Figure 7G). RT‐qPCR validation demonstrated significant upregulation of *FgCYP637A1* under Teb treatment in wild‐type PH‐1, whereas its expression was substantially attenuated in Δ*FgSR* transformants (Figure 7H). Yeast one‐hybrid assays further verified that FgSR directly bound to the DRE motif in the promoter region of *FgCYP637A1* (Figure 7I). As expected, knockout transformant Δ*FgCYP637A1* displayed significantly increased Teb sensitivity compared to wild‐type PH‐1 (Figure 7J). Similarly, molecular docking revealed that FgCYP637A1 has a high binding affinity for Tri, with a binding free energy of −7.176 kcal mol^−1^ and the knockout transformant Δ*FgCYP637A1* exhibited increased sensitivity to Tri (Figure 7J and Figure S6I). These collective findings demonstrate that FgCYP637A1 is a key determinant of DMI sensitivity in *F. graminearum*, executing detoxification roles functionally comparable to MoDde1 in *M. oryzae*.

### 2.10 Lowering MoDde1 Gene Expression via Small‐molecule Compound D1 Application and Gene Silencing in Transgenic Rice Line HIGS‐MoDde1 Increased Susceptibility to DMI Fungicides in Rice Blast Control

**FIGURE 8:**
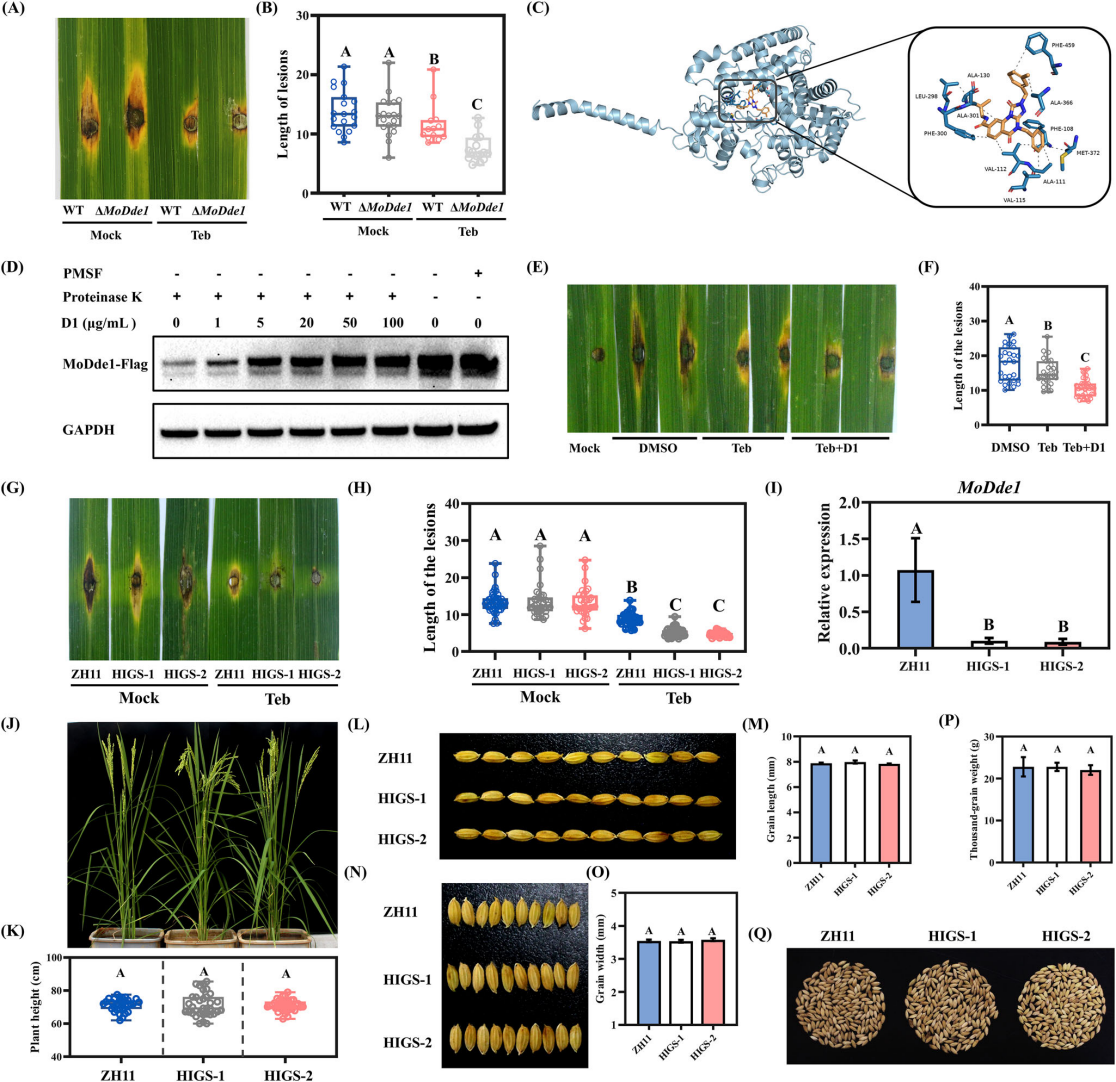
The utilization of MoDde1 protein inhibitors or the HIGS‐MoDde1 rice lines in conjunction with Teb applications for the effective management of rice blast disease. (A) The DMI fungicide Teb effectively controlled the rice blast caused by the Δ*MoDde1* strain. (B) Statistical data on lesion length of Δ*MoDde1* under DMI treatment. Data are the mean ± SD (*n* >10). (C) AutoDock Vina was used to perform molecular docking between MoDde1 and inhibitor D1. (D) The interaction between MoDde1 and inhibitor D1 was verified using DARTS. The protease was used at a concentration of 1:1000, while the concentrations of D1 were 1, 5, 20, 50 and 100 µg mL^−1^. (E) Combination of D1 and Teb enhanced the control efficiency against rice blast. Concentrations of Teb and D1 were 5 and 10 µg mL^−1^, respectively. (F) Statistical analysis demonstrated that the combined use of Teb and D1 led to a significant reduction in lesion length of rice blast. Data are the mean ± SD (*n* >25). (G) The HIGS‐MoDde1 rice lines enhanced the control efficiency of Teb against rice blast. (H) Statistical analysis was performed to compare the lesion lengths of rice blast in HIGS‐MoDde1 rice lines with and without Teb treatment. Data are the mean ± SD (*n* >30). (I) The expression level of *MoDde1* in *M. oryzae* was determined by RT‐qPCR when the fungus was inoculated into HIGS‐MoDde1 rice lines. Data were normalized to *MoActin* and presented as the mean ± SD (*n* = 3). (J, L, N, Q) The HIGS‐MoDde1 rice lines did not show differences from parental rice ZH11 for plant height, grain length, grain width, and 1000‐grain weight of rice (K, M, O, P). Statistical analysis was conducted for the plant height, grain length, grain width, and 1000‐grain weight, no significant differences were observed between ZH11 and the HIGS‐MoDde1 rice lines. Statistical analyses of Figure 8B,F,H,I,K,M,P, and O were conducted via the LSD method, where values marked with identical letters show no significant difference, with uppercase letters denoting significance at *p* = 0.01.

Since MoDde1 is a key factor determining the DMI sensitivity in *M. oryzae*, we investigated whether suppression of MoDde1 can increase the control efficacy of DMI fungicides in *M. oryzae*. Results indicated that inhibiting *MoDde1* expression significantly enhanced the control efficacy of DMI fungicides against rice blast (Figure 8A,B), providing a clear molecular target for developing next‐generation DMI synergism strategies. Therefore, we developed two innovative control tools targeting MoDde1, i.e., a specific small‐molecule inhibitor and host‐induced gene silencing (HIGS) rice lines.

To efficiently screen for small‐molecule inhibitors targeting MoDde1, we established a tiered screening pipeline encompassing four sequential stages: target validation, virtual screening, in vitro activity evaluation, and in vivo efficacy verification. Following the screening of 550 000 small‐molecule compounds based on this pipeline, we performed activity validation on the top 5 candidates with the highest comprehensive performance (Figure S8A). Among these assays, the plate inhibition test confirmed that the small‐molecule inhibitor compound D1 (chemical name: 4‐benzyl‐N‐isobutyl‐2‐(3‐methylbe‐nzyl)‐1,5‐dioxo‐1,2,4,5‐tetrahydro‐[1,2,4]triazolo[4,3‐a]quinazoline‐8‐carboxamide, molecular formula: C_29_H_29_N_5_O_3_) could effectively enhance the fungicidal activity of the DMI fungicide Teb even at low concentration (0.3 µg mL^−1^) (Figure S8B,C). Combined results from molecular docking simulations and in vitro DARTS validation assays further confirmed the specific binding interaction between the target protein MoDde1 and the small‐molecule inhibitor D1 (Figure 8C,D). Further yeast heterologous expression assays demonstrated that D1 significantly reduced the Teb resistance of yeast expressing *MoDde1*, whereas D1 treatment did not alter Teb sensitivity in yeast expressing *MoCYP51A* or *FgCYP637A1*, indicating that D1 specifically inhibits MoDde1 activity (Figure S8D). In addition, we found that D1 exhibits a narrow inhibitory spectrum and fails to enhance the antifungal activity of Teb against *F. graminearum* PH‐1 (Figure S8E). Controlled greenhouse trials for rice blast management demonstrated that co‐application of inhibitor D1 and the fungicide Teb enhanced the overall control efficacy by nearly 25%, and no antagonistic effects were detected between the two compounds (Figure 8E,F and Figure S8F).

We also constructed a HIGS vector expressing *MoDde1*‐specific hairpin RNA (Figure S8G) and successfully generated the transgenic rice line HIGS‐MoDde1. Pot trials demonstrated that HIGS‐MoDde1, when combined with Teb treatment, improved control efficacy by 20%–30%, significantly outperforming the non‐transgenic control (Figure 8G,H and Figure S8H). Molecular analysis confirmed that this line effectively silenced the expression of *MoDde1* upon pathogen infection (Figure 8I). Moreover, key agronomic traits including plant height, grain shape, and thousand‐grain weight showed no significant differences compared to the wild‐type control (Figure 8J,Q), indicating the high potential for field application.

In summary, the two MoDde1‐targeted strategies developed in this study achieve effective control while reducing Teb application by more than 20%. This approach provides theoretical innovation and technical support for the green and integrated management of rice blast, demonstrating significant scientific value and promising application prospects.

## 3 Discussion

DMIs, including azole antifungals, specifically target the fungal cytochrome P450 enzyme 14α‐demethylase (CYP51). This inhibition blocks the demethylation of lanosterol, disrupting ergosterol biosynthesis, a process essential for fungal membrane integrity [18]. Concurrently, the accumulation of toxic sterols and activation of autophagy pathways synergistically compromise cellular homeostasis, ultimately driving fungal pathogen death [6]. Pathogenic fungi often enhance their resistance to DMIs through alterations or overexpression of the *CYP51* gene, and overexpression of ABC or MFS transporter‐encoding genes [21, 22, 23].

Transcription factors involved in ergosterol synthesis are essential in modulating DMI sensitivity. Notably, the Zn_2_Cys_6_ transcription factors frequently assume a significant role in this regulatory process in pathogenic fungi. In *S. cerevisiae*, ScUpc2 and ScPdr1/ScPdr3 participate in the regulation of DMI sensitivity by regulating the expression of downstream genes *ScERG11* and *ScPdr5*, respectively [24, 25, 26]. In *B. cinerea*, BcMrr1 activates the overexpression of the downstream ABC transporter‐encoding gene *BcAtrB*, leading to multidrug resistance [27]. In *S. homoeocarpa*, ShXDR1 activates the overexpression of ABC transporter‐encoding genes *ShPdr1* and *ShAtrD*, leading to MDR [28]. In *A. fumigatus*, AfAtrR targets *AfCYP51* and the ABC transporter protein‐encoding gene *AfCdr1B* to regulate DMI sensitivity [15, 29]. In *F. graminearum*, FgSR targeting the ergosterol synthesis pathway genes *FgCYP51A*, *FgCYP51B* and *FgCYP51C* is involved in the regulation of DMI sensitivity, and this mechanism is conserved among the Sordariomycetes and Leotiomycetes fungi [11, 30, 31, 32].

In this study, SR has a differential regulatory effect on the DMI sensitivity in Sordariomycetes fungi and Leotiomycetes fungi. Specifically, SR has a stronger regulatory effect on the DMI sensitivity in Sordariomycetes fungi, while SR has a weaker regulatory effect on the DMI sensitivity in Leotiomycetes fungi. Through in‐depth research on the mechanism of MoSR in *M. oryzae*, it is revealed that MoSR not only targets the genes *MoCYP5A*, *MoCYP5B*, *MoERG6A*, and *MoERG6C*, which are implicated in ergosterol biosynthesis, but also engages with the DRE motif located within the promoter region of *MoDde1*, which was relatively conserved cytochrome P450 enzyme‐encoding gene in Sordariomycetes fungi. It is consistent with the observations of the difference in EC_50_ values between the *ΔMoSR‐OEMoDde1* and OE*MoDde1* strains. In the Δ*MoSR*‐OE*MoDde1* strain, only *MoDde1* was overexpressed alone. Due to the deletion of *MoSR*, the expression levels of sterol biosynthesis‐related genes including *CYP51A* and *CYP51B* could not be upregulated normally. In contrast, the complete SR‐mediated regulatory network was retained in the OE*MoDde1* strain, which could coordinately regulate the expression of downstream resistance‐related genes such as *CYP51A* and *CYP51B*. Such a regulatory difference ultimately resulted in significantly lower Teb EC_50_ values in Δ*MoSR*‐OE*MoDde1* than those in OE*MoDde1*. The cytochrome P450 enzyme is a heme protein commonly used for the modification of endogenous substrates (fatty acids, sterols, bile acids, etc.) and the metabolism of exogenous substances (drugs, plant secondary metabolites, organic pollutants, etc.) [33]. The cytochrome P450 enzyme is the main component of the drug metabolism enzyme system, which is rapidly activated and expressed upon the appearance of exogenous compounds, minimizing potential damage [34]. The metabolic detoxification resistance mechanism mediated by overexpression of cytochrome P450 enzymes is common in insecticide or herbicide resistance, with few reports on fungicide resistance [35, 36]. In *M. oryzae*, transcription factors MoVelB or MoIRR can activate the expression of downstream cytochrome P450 enzyme‐encoding genes, enhancing the isoprothiolane cytotoxicity [37, 38]. In the process of pathogen evolution and gene expansion, many pathogens have unique cytochrome P450 enzymes within their genus or species, endowing them with unique metabolic modification abilities [39]. In the genus *Aspergillus*, the cytochrome P450 enzyme AsBapA (CYP617D1) specifically degrades benzo [a] pyrene, which is an organic pollutant [40]. In this study, we found that MoDde1 represents a drug‐metabolizing enzyme whose orthologs are conserved among Sordariomycetes fungi. This enzyme is transcriptionally induced upon DMI exposure and functions to detoxify these compounds. We have not yet identified the metabolites of DMI fungicides in this work, which is a limitation of the present study, and we will conduct dedicated research on this issue in the future. Combined with the results of molecular docking and DARTS assays, we conclude that MoDde1 can differentially bind and metabolize DMI fungicides including Teb (strong) and Tri (weak).

Variations in the DRE motif of the promoter prevent the expression of the *Dde1* genes. For instance, *FgDde1* could not be induced by DMIs because of the mutation of TA to AC in the DRE motif. The direct binding of FgSR to the DRE motif has been previously confirmed by electrophoretic mobility shift assay (EMSA) (Liu et al., 2019), supporting the regulatory role of this cis‐element in mediating DMI‐inducible expression. Nevertheless, the functional deficiency of *FgDde1* is compensated by another target gene of FgSR, namely *FgCYP637A1*, which contains the intact DRE motif (e.g., 5’‐CGAATACGA‐3’) in its promoter region. The *MoDde1* homologous gene only exists in some Sordariomycetes fungi, and it is not clear how Sordariomycetes fungi efficiently regulate the DMI sensitivity without the corresponding *Dde1* gene.

A series of fungus‐targeted small‐molecule inhibitors have been discovered in previous studies. The natural product natamycin directly binds to FfSR and inhibits its phase separation and transcriptional activity, thereby significantly enhancing antifungal efficacy when combined with prochloraz and other azole fungicides [32]. Small molecules F2058‐0186 and STOCK2S‐9031 interact with FolIws1, block the formation of the FolIws1‐FolTFIIS protein complex, and strongly suppress conidiation of *F. oxysporum* f. sp. *Lycopersici* [41]. In addition, the lead compound Pan‐RAS‐IN‐1 specifically targets MoCox6, an inner mitochondrial membrane protein of *M. oryzae*. It sequentially disrupts protein modification and molecular interactions, blocks mitophagy, and ultimately inhibits the vegetative growth and pathogenicity of the fungus [42]. The small‐molecule inhibitors alexidine dihydrochloride [43], propranolol [44], UGE1i [45] and Diphenyl ether ester compound FY2100 [46] target phosphatidylglycerol synthase MoGep4, phosphatidate phosphatase Pah1, UDP‐glucose 4‐epimerase MoUGE1 and toxic effector MoErs1, respectively. All of them can significantly block the infection process of *M. oryzae*.

Through virtual screening and in vitro validation, we identified D1, a specific small‐molecule inhibitor of MoDde1. This compound targets the active site of MoDde1 and effectively inhibits its function, providing a valuable tool for enhancing the efficacy of DMI fungicides. D1 exhibits synergistic effects with Teb for rice blast control, allowing Teb rates to be reduced while maintaining comparable control efficacy, which may minimize pesticide residues and delay resistance development. However, the application of D1 still requires optimization. From both cost and environmental perspectives, the current formulation has not achieved an ideal “synergism‐reduction” balance. This may be attributed to the following factors, e.g., as a lead compound, D1 still could be improved in its binding efficiency with the target; its pharmacokinetic properties, such as permeability and stability on plant surfaces, have not been systematically optimized, potentially leading to insufficient duration of effective concentration at the action site; moreover, D1 lacks direct fungicidal activity and relies entirely on inhibiting MoDde1, making it alone incapable of controlling disease. Based on these findings, future structural optimization of D1 can focus on three aspects. First, based on the structural information of the MoDde1‐D1 complex, key active regions could be modified to enhance binding affinity, with the parallel aim of exploring the introduction of direct antifungal activity, employing a strategy analogous to the activity‐enhancing modifications used in melatonin‐based compounds [47]; Second, improve water solubility and leaf retention, and consequently, bioavailability, either by introducing hydrophilic groups into the molecular structure or by formulating the compound with surfactants; Third, rationally integrate the active pharmacophores of D1 and Teb to design a single chemical entity that concurrently targets both MoDde1 and CYP51. This chimera strategy, inspired by dual‐functional agents like the mTOR inhibitor/GSPT1 degrader YB‐3‐17, aims to develop a more robust and simplified control regimen with enhanced practical utility [48].

**FIGURE 9:**
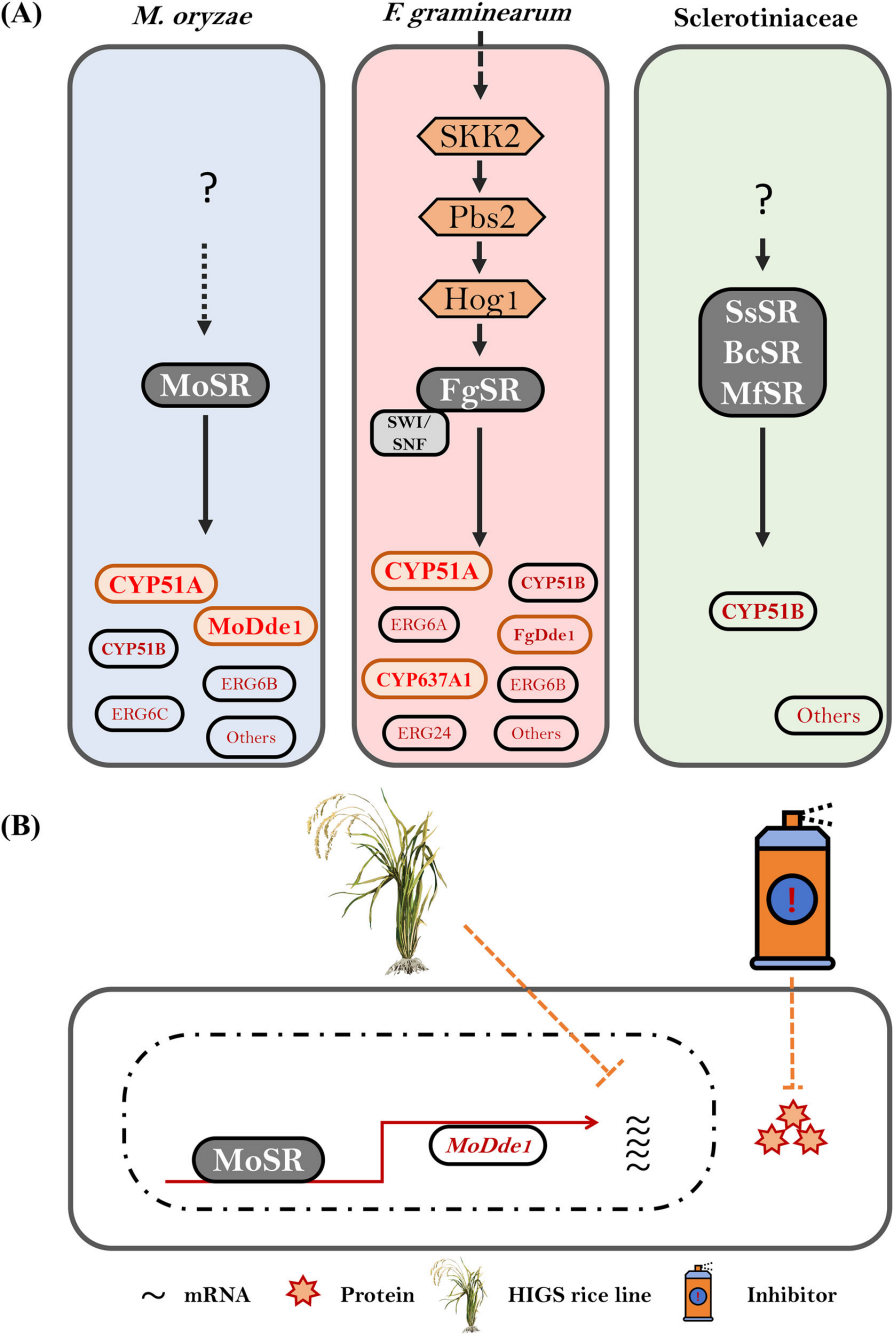
Mechanisms of MoSR‐mediated regulation for DMI sensitivity via transcriptional control of *MoCYP51A* and *MoDde1* in *M. oryzae*, and green control strategies for rice blast. (A) Mechanisms underlying differential regulation of DMI fungicide sensitivity by SR in Sordariomycetes fungi (*M. oryzae*, *F. graminearum*) and Sclerotiniaceae fungi (*S. sclerotiorum*, *M. fructicola*, *B. cinerea*). (B) Strategies for developing MoDde1 protein inhibitors and *MoDde1* mRNA‐targeted HIGS rice lines.

In conclusion, as shown in Figure 9, we identified a novel cytochrome P450 enzyme, MoDde1, regulated by MoSR; the MoSR/MoDde1 regulatory axis plays a critical role in modulating the DMI sensitivity in *M. oryzae*. SR exhibits differential regulation of DMI sensitivity in Sordariomycetes and Leotiomycetes fungi by targeting varying quantities of downstream essential genes. In addition, we designed a MoDde1‐related small molecule inhibitor D1 and host‐induced gene silencing in rice, providing methods and materials for future control of rice blast and reduction of DMI usage.

## 4 Experimental Section

### 4.1 Strains, Media and Fungicides

The *M. oryzae* strains including wild‐type isolate H08‐1a and transformants, are listed in Table S1. The *F. graminearum* wild‐type isolate PH‐1, *B. cinerea* wild‐type isolate B05.10, *Monilinia fructicola* wild‐type isolate ZFTF5, *Sclerotinia sclerotiorum* wild‐type isolate 1980s and their associated SR knockout transformants are also listed in Table S1 [32, 49]. All strains were cultured on potato dextrose agar (PDA, 200 g Potato, 20 g Dextrose, 20 g agar, and add water to 1 L) medium for 5 days at 27°C in the dark [50]. For vegetative growth, 3 mm × 3 mm mycelial plugs from the periphery of freshly cultured strains were inoculated onto media CM (6 g NaNO_3_, 0.52 g KCl, 0.52 g MgSO_4_·7H_2_O, 1.52 g KH_2_PO_4_, 10 g Dextrose, 1 mL Trace element and 1 mL Vitamin solution, and add water to 1 L), PDA or Tomato Oat Agar (OTA, 150 mL tomato juice, 40 g Oats, 0.6 g CaCO_3_, 20 g agar, and add water to 1 L). The DMI fungicide tebuconazole (Teb) was dissolved in acetone to make a stock solution at a concentration of 2000 µg a.i. /mL. Sensitivity to Teb was assessed on PDA amended with tebuconazole (Teb) at 0, 0.001, 0.05, 0.1, 0.2, 0.3, 0.4 and 0.8 µg mL^−1^. Sensitivity to fludioxonil (FLU), iprodione (IPR), Isoprothiolane (IPT), carbendazim (CAR), Azoxystrobin (AZO) and boscalid (BOS) was assessed on PDA amended with corresponding fungicides at concentrations of 4, 20, 5, 0.3, 1, 30 µg mL^−1^, respectively. Sensitivity to Triadimefon (Tri), Propicoazole (Prop), Prochlorazole (Proc), Diniconazole (Din), Ketoconazole (Ket) and Econazole (Eco) was assessed on PDA amended with corresponding fungicides at concentrations of 1, 0.6, 0.025, 0.2, 0.5, 0.8 µg mL^−1^, respectively.

### 4.2 Phylogenetic and Amino Acid Conservation Analysis of SR Protein

The amino acid sequences of the SR and BLAST Servers at NCBI from the *Magnaporthe* genome (https://www.ncbi.nlm.nih.gov/genome/51706) were used. Construction of phylogenetic trees was based on amino acid sequences of the SR in *M. oryzae*, *B. cinerea*, *M. fructicola*, *S. sclerotiorum*, *Trichoderma reesei*, *Ustilaginoidea virens*, *Colletotrichum graminicola*, *Neurospora crassa*, and four *Fusarium* species. Phylogenetic trees were constructed by comparing the identified amino acid sequences using the neighbor‐joining method (number of bootstrap replications was 1000) in MEGA7.0. SR amino acid sequence comparison was performed using the software BioEdit.

### 4.3 Genetic Manipulations Including Knockout, Complementation and Overexpression

To clarify the differences of SR function in Sordariomycetes and Leotiomycetes fungi, we knocked out the *FgSR* (*FGSG_01176*) in *F. graminearum*, the *MoSR* (*MGG_14728*) in *M. oryzae*, the *BcSR* (*BCIN_02g03500*) in *B. cinerea*, the *SsSR* (*SG1G_01701*) in *S. sclerotiorum*, and the *MfSR* (*EYC84_005341*) in *M. fructicola*. Double‐joint PCR was used to generate the knockout constructs of *SR*. To generate complemented transformants of the Δ*MoSR* knockout transformant, a full *MoSR* genomic region, including its upstream 1.5‐kb region, was inserted into the plasmid pGTN for transformation. The strains for heterologous expression of *MfSR* in the MoSR knockout mutant were constructed using the plasmid pTNHG‐MfSR, which harbors the full‐length coding sequence of *MfSR* driven by the 1.5‐kb promoter fragment of *MoH3* (*MGG_01159*). Genetic transformation was conducted by using PEG‐mediated protoplast transformation [37, 38].

Evaluation of stress sensitivity: To test sensitivity of strains against different stresses, mycelial growth was assayed after incubation at 27°C for 5 days on PDA plates and PDA amended with 0.7 M NaCl, 0.8 M KCl, 1.2 M sorbitol (SOR), 0.025% SDS (w/v), 1200 µg mL^−1^ Congo red (CR), 1200 µg mL^−1^ calcofluor white (CFW) and 6 mM H_2_O_2_, respectively.

### 4.4 Conidiation, Conidial Germination, Appressorium Formation and Pathogenicity Test

A plug with mycelia and conidia at about 1 cm^2^ grown on OTA plates for 7 days was taken and dissolved in 1 mL of water, and conidia were counted with hemocytometer. Next, 15 µL aliquots of the spore suspension were dropped on sterilized plastic coverslips and incubated in a humid chamber at 25°C under dark conditions. After incubation for 4 and 24 hpi, conidial germination and appressorium formation of 50 conidia were investigated, respectively. For the pathogenicity test, plugs of 3‐mm mycelium from wild‐type H08‐1a and the mutants were inoculated on rice ZH11 leaves for 10 days, and spot lengths were measured.

### 4.5 RNA Preparation and RT‐qPCR

Mycelia from the relevant strains were collected under the specific conditions and times, frozen rapidly in liquid nitrogen, and stored at ‐80°C until use. Total RNA isolation was conducted by using TRIzol (YEASEN Biotech Co., Ltd, Wuhan, China). cDNA was prepared using a HifairII first Strand cDNA Synthesis kit (YEASEN Biotech Co., Ltd) with oligo (dT). Reverse transcription quantitative PCR (RT‐qPCR) was performed with ChamQTM SYBR qPCR Master Mix (Vazyme Biotech Co., Lth) on a Bio‐Rad CFX96 real‐time PCR detection system. The comparative cycle threshold (CT) method was used for data analysis, and relative fold difference was expressed as 2^−ΔΔCT^ [51]. As an internal reference, *MoActin* was used for each quantitative real‐time PCR analysis. Primer sequences used are shown in Table S2.

### 4.6 RNA Sequencing

RNA sequencing was conducted on the Illumina HiSeq 4000 PE150 platform using 150 bp paired‐end libraries with 500 bp inserts at Wuhan SeqHealth Technology Company. Transcriptome data quality was controlled using fastp (version 0.23.0), and over 35 million high‐quality reads per sample were achieved. The RPKM (Reads per Kilobase per Million Reads) value was used as a measure of gene expression; the gene was considered a differentially expressed gene when log_2_ (FoldChange (Δ*MoSR*_RPKM/H08‐1a_RPKM)) > 1 or < ‐1 and *p*‐value < 0.05 [52]. KEGG and GO analyses were performed using the DAVID Bioinformatics Resources online website (https://david.ncifcrf.gov/home.jsp) [53].

### 4.7 Yeast One‐Hybrid Analysis

The potential interaction between MoSR and the promoters of *CYP51A*, *CYP51B* and *MoDde1* was verified by using the Y1H assay. The promoter sequences of *CYP51A*, *CYP51B* and *MoDde1* were amplified and inserted between the *Eco*RI and *Spe*I sites of the pHIS2 vector. The full‐length cDNA sequence of *MoSR* was inserted into the *Eco*RI site of the pGADT7 vector. The plasmid pairs of pHIS2‐*CYP51A*‐p, *MoDde1*‐p, or *MoDde1*‐p / pGADT7‐MoSR were co‐transformed into Y187 using the LiAc/Carry‐DNA/PEG3350 transformation method. 3AT was used at a concentration of 40 mM. The plasmid pair of pHIS2‐53/pGADT7‐53 served as the positive control.

### 4.8 Dual Luciferase Assay

The full‐length cDNA sequence of MoSR was cloned into the pGreen II 62SK vector, and the 50 bp promoter of *MoDde1* gene containing DRE motif was cloned in pGreen II 0800‐Luc vector, and the recombinant vectors were transformed into Agrobacterium GV3101 containing P19 plasmid. Agrobacteria containing the pGreen II 62SK‐MoSR or pGreen II 0800‐MoDde1‐p‐Luc vector were mixed and injected into tobacco leaves at a ratio of 1:1. Two to three days later, they were observed by plant live imaging (NightSHADE L985, Berthold, Germany), and the pGreen II 62SK‐empty and pGreen II 0800‐MoDde1‐p‐Luc vectors were used as negative controls.

### 4.9 Determination of Ergosterol Content

The wild‐type and mutant strains were cultured in PDB for 48 h and subsequently treated with 0.5 µg mL^−1^ Teb for 3 h. Mycelial samples, each weighing 0.5 g, were collected and mixed with a NaOH‐methanol solution. The mixture was subjected to saponification in a water bath at 80°C for 1 h. Then the solution was concentrated through petroleum ether distillation and re‐suspended in 10 mL of a methanol‐chloroform solution (1:1, V/V). The resulting solution was filtered using a 0.45 µm organic membrane filter, and the ergosterol content was analyzed using a high‐performance liquid chromatography (HPLC) system (Shimadzu LC‐20A).

### 4.10 High‐Throughput Screening of Small‐Molecule Inhibitors for MoDde1

First, the complete amino acid sequence of MoDde1 was retrieved from the *M. oryzae* genome database in Ensembl. Its three‐dimensional structure was predicted using AlphaFold3 under the default “monomeric protein high‐accuracy mode,” and the initial model was refined based on homology sequence conservation analysis. Subsequently, molecular docking was performed using AutoDock 4.2 with PyMOL for visualization, employing known DMI fungicides (e.g., Teb) as probe molecules. By defining potential binding regions and analyzing key interactions including hydrogen bonding, hydrophobic packing, and salt bridge formation, the core active site for DMI binding in MoDde1 was precisely identified. For virtual screening, the validated MoDde1 active site was used as the receptor, and small molecules from the Life Chemicals compound library were used as ligands. High‐throughput rigid docking was conducted targeting the binding pocket within the active site. Compounds were initially ranked using the AutoDock scoring function, from which the top 50 compounds with binding energies ≤ –7 kcal/mol were selected. These candidates were further evaluated using SwissADME and ProTox‐II to predict their ADMET (Absorption, Distribution, Metabolism, Excretion, and Toxicity) properties. Finally, five candidate MoDde1 inhibitors exhibiting high binding affinity and favorable pharmacokinetic profiles were identified for subsequent commercial procurement (purity ≥ 98%).

### 4.11 Development of HIGS‐MoDde1 Rice Lines

A 453 bp fragment of the coding sequence (CDS) of *MoDde1* from its 3′‐terminus was selected as the RNAi target, and the corresponding inverted repeat was cloned into the binary vector DS1301 to generate the hairpin construct pDS1301‐HIGS‐MoDde1 [54, 55]. Rice transformation was carried out via *Agrobacterium*‐mediated delivery into cultivar ZH11 (performed by Biorun Biotechnology Co., Ltd., Wuhan, China). Transgenic plants were selected on hygromycin and verified by PCR using construct‐specific primers. After successive propagation through T_0_, T_1_, and T_2_ generations, homozygous HIGS‐MoDde1 lines (T_2_) were established. To assess gene silencing efficiency, homozygous and wild‐type control plants were inoculated with *M. oryzae*. At 48 h post‐inoculation, fungal RNA was extracted from infected leaf tissues, and expression of MoDde1 was quantified by RT‐qPCR using *MoActin* as the endogenous reference.

### 4.12 Darts

The MoDde1‐Flag‐expressing strain was cultured in PDB medium for 48 h, and the mycelia were collected for total protein extraction. The protein samples were divided into equal‐volume aliquots and incubated with small‐molecule compounds or fungicides at different concentrations to facilitate their binding. Subsequently, proteinase K was added for digestion. The target protein bound to small molecules or fungicides exhibited stable conformation and resistance to enzymatic cleavage. After termination of digestion, the samples were separated by SDS‐PAGE and subjected to Western blot analysis using anti‐Flag antibody. The binding capacity between MoDde1 and small molecules or fungicides was determined according to the differences in protein bands.

### 4.13 Statistical Analysis

Raw data were preliminarily sorted and processed, and outliers were eliminated according to experimental rules. All experimental results were expressed as mean ± standard deviation (mean ± SD). The sample size of each group was clearly marked in the figure legends. Significant differences were analyzed by independent‐samples t‐test or one‐way analysis of variance (ANOVA), followed by least significant difference (LSD)’s multiple range test for multiple comparisons. The significance level was set at *p* < 0.05. All statistical analyses were conducted using SPSS 17.0 software, and all graphs were constructed using GraphPad Prism 8.0 software.

Schematic diagrams: Schematic diagrams were drawn with WPS Office, and the rice images presented in the figures were obtained from Bioicons. Rice icon by DBCLS https://togotv.dbcls.jp/en/pics.html is licensed under CC‐BY 4.0 Unported https://creativecommons.org/licenses/by/4.0/.

## Author Contributions

**F.M**. and **C.L**. conceived and designed the study. **F.M**., **W.W**., and **M.W**. performed experiments. **F.M**., **M.C**., **Y.W**., **L.Z**., and **S.M**. collected and analyzed the data. **F.M**., **Y.W**., **S.M**., **L.Y**., **W.Y**., **G.S**., and **C.L**. wrote and edited the paper. All authors reviewed and approved the final version for publication.

## Conflicts of Interest

The authors declare no conflicts of interest.

## Supporting information




**Supporting File**: advs77796‐sup‐0001‐SuppMat.docx.

## Data Availability

The data that support the findings of this study are available from the corresponding author upon reasonable request.
