# Supplementary material for: The MoSR‐MoDde1 Regulatory Axis: A Novel Determinant of DMI Sensitivity and a Target for Sustainable Rice Blast Control
Source: Adv Sci (Weinh). 2026 Sep 16:e77796. Online ahead of print. doi: 10.1002/advs.77796 (PMC13583232; doi:10.1002/advs.77796)
Supplement: Supplementary file 1 [file ADVS-9999-e77796-s001.docx]

Supplementary files

**Table S1.** ***Magnaporthe oryzae* strains used in this study**

| **Strains** | **Description** | **Reference** |
| --- | --- | --- |
| H08-1a | M. *oryzae* wild type strain H08-1a | Wang et al., 2018 |
| Δ*MoSR* | *MoSR* (MGG_14782) knockout transformant | This study |
| PH-1 | *F. graminearum* wild type strain PH-1 | This study |
| Δ*FgSR* | *FgSR* (*FGSG_01176*) knockout transformant | This study |
| B05.10 | *B. cinerea* wild type strain B05.10 | This study |
| Δ*BcSR* | *BcSR* (*BCIN_02g03500*) knockout transformant | This study |
| 1980s | *S. sclerotiorum* wild type strain 1980s | This study |
| Δ*SsSR* | *SsSR (SS1G_01701*) knockout transformant | Zhao et al., 2025 |
| ZYTF5 | *M. fructicola* wild type strain ZFTF5 | This study |
| Δ*MfSR* | *MfSR (EYC84_005341*) knockout transformant | This study |
| Δ*MoDde1* | *MoDde1* (*MGG_01391*) knockout transformant | This study |
| Δ*MoDde1*-C | *MoDde1* complemented transformant with 2k native promoter and full length of *MoDde1* in the background of Δ*MoDde1* | This study |
| OE*MoDde1* | The *MoH3* (*MGG_01159*) 1.5 kb promoter with the *MoDde1* cds | This study |
| Δ*MoCYP51A* | *MoCYP51A* (*MGG_04628*) knockout transformant | This study |
| Δ*MoCYP51B* | *MoCYP51B* (*MGG_04432*) knockout transformant | This study |
| Δ*MoERG6A* | *MoERG6A* (*MGG_10508*) knockout transformant | This study |
| Δ*MoERG6C* | *MoERG6C* (*MGG_10806*) knockout transformant | This study |
| Δ*MoSDR13* | *MoSDR13* (*MGG_07793*) knockout transformant | This study |
| Δ*MoCYP51A*  Δ*MoDde1* | *MoCYP51A* and *MoDde1* double knockout transformant | This study |
| Δ*MoSR-OEMoDde1* | *MoDde1* was overexpressed in the Δ*MoSR* knockout transformants. | This study |
| Δ*MoDde1*-C-ΔDRE | Transformants of MoDde1 with a DRE motif-deficient promoter | This study |
| *pTrpc-FgDde1* | The *FgDde1* gene (*FGSG_11536*) was expressed in *F. graminearum* under the control of the *pTrpC* promoter. | This study |
| *pEF1-MoDde1* | The *MoDde1* gene was expressed in *B. cinerea* under the control of the *pEF1* promoter. | This study |
| Δ*FgDde1* | *FgDde1* knockout transformant | This study |
| Δ*FgCYP637A1* | *FgCYP637A1* (*FGSG_03498*) knockout transformant | This study |
| HIGS-MoDde1 | *MoDde1* silencing rice line | This study |

| **Table S2. Primers used in this study** | | |  |
| --- | --- | --- | --- |
| **Name** | **Sequence (5′-3′)** | **Primer description** | |
| DJ1-*MoSR*-5F | CCTTCCGACGCCTAGAAATC | *MoSR* knockout primers | |
| DJ1-*MoSR*-5R | TCAATATCATCTTCTGTCGACGCAGGATCATAAGGTGGTT |  |  |
| DJ1-*MoSR*-3F | AGATGCCGGATCCACTTAACAAAGATTTAGCTGCGAGTTG |  |  |
| DJ1-*MoSR*-3R | CCAGACTGAAGTACTGCCCAA |  |  |
| DJ3-*MoSR*-F | ATGCCGAAAAATCTACGGGAAA |  |  |
| DJ3-*MoSR*-R | AAGCCTGCCTTTCTTTACAT |  |  |
| *MoSR*-Check-F | ATGCCGCCGCGACGCTCA | Validation primers for *MoSR* knockout transformants | |
| *MoSR*-Check-F | TCAAGAGCTGCTTCGGGCAG |  |  |
| DJ1-*FgSR*-5F | TCTCATGTTCATCTGCCGGA | *FgSR* knockout primers | |
| DJ1-*FgSR*-5R | TCAATATCATCTTCTGTCGAGGAGAGAACCCTAGATCGTG |  |  |
| DJ1-*FgSR*-3F | AGATGCCGGATCCACTTAACTTTGAGAGCTGGTCTCGAGG |  |  |
| DJ1-*FgSR*-3R | TCTTTGGCAGCAGCCAGAAG |  |  |
| DJ3-*FgSR*-F | CTCTCATTCCCAGGGTCTCT |  |  |
| DJ3-*FgSR*-R | TGATGAGCGCACCAAGAAGA |  |  |
| *FgSR*-Check-F | ATGCCGCCTCGTCGGTCTCA | Validation primers for *FgSR* knockout transformants | |
| *FgSR*-Check-R | TTAGTCGGGGGAATCAGGCA |  |  |
| DJ1-*BcSR*-5F | GACAGTCATTTTGGAGGGGT | *BcSR* knockout primers | |
| DJ1-*BcSR*-5R | TCAATATCATCTTCTGTCGAGGTGGCGACCAAAATGACTA |  |  |
| DJ1-*BcSR*-3F | AGATGCCGGATCCACTTAACCGGAATAGAGCTGGTCTCGAGG |  |  |
| DJ1-*BcSR*-3R | AGGAGGACCTTGGTTCTATT |  |  |
| DJ3-*BcSR*-F | TAAGAAGAGGCAGGAGGGTT |  |  |
| DJ3-*BcSR*-R | CCCCTGCCACAAAGCTTAAA |  |  |
| *BcSR*-Check-F | ATGCCACCAAGAAGATCGCA | Validation primers for *BcSR* knockout transformants | |
| *BcSR*-Check-R | TTAGTCTGGTGGCGGTGC |  |  |
| DJ1-*MfSR*-5F | CGCAATGTCTATCACACCGG | *MfSR* knockout primers | |
| DJ1-*MfSR*-5R | CTCCTTCAATATCATCTTCTGTCGACTTTGACATCCCGCTTTGGAC |  |  |
| DJ1-*MfSR*-3F | GATGCCGGATCCACTTAACCATGCGCAAGTTATAGAGAATG |  |  |
| DJ1-*MfSR*-3R | GCTAGATCTACGGACGCTGC |  |  |
| DJ3-*MfSR*-5F | GTTGATTCCGGAGGTCCGT |  |  |
| DJ3-*MfSR*-3R | CCATAGGGAGTCGATAGAGTCG |  |  |
| *MfSR-*Check-F | CCATTACATCAATCGCCTATGCA | Validation primers for *MfSR* knockout transformants | |
| *MfSR-*Check-R | TCGTAGCCACTGCCAGGAA |  |  |
| DJ1-*MoDde1*-5F | CTTTTGGTGCTACAACCTTGA | *MoDde1* knockout primers | |
| DJ1-*MoDde1*-5R | TCAATATCATCTTCTGTCGATTTCCCCCTTCTGATGCAGT |  |  |
| DJ1-*MoDde1*-3F | AGATGCCGGATCCACTTAACCATCTTTGTCGGCATGGCAT |  |  |
| DJ1-*MoDde1*-3R | TTGGCTTTCGGGAAAGTGCA |  |  |
| DJ3-*MoDde1*-F | GTATCAACAGTAGCGCCACG |  |  |
| DJ3-*MoDde1*-R | GCAAAGCGTCTTGACCAGGT |  |  |
| *MoDde1*-Check-F | ATGTCGACGACGCAACTCG | Validation primers for *MoDde1* knockout transformants | |
| *MoDde1*-Check-R | TCAAGCACGCTCCTTGAAAA |  |  |
| DJ1-*ERG6A*-5F | GTTGTTCTGCGCAAAGAATT | *ERG6A* knockout primers | |
| DJ1-*ERG6A*-5R | TCAATATCATCTTCTGTCGAGAGGACTTTTGGTTGTAAAGGA |  |  |
| DJ1-*ERG6A*-3F | AGATGCCGGATCCACTTAACGACGGGATTGGTATTCTTTGA |  |  |
| DJ1-*ERG6A*-3R | GCGAAGTTGTACGTCATGAA |  |  |
| DJ3-*ERG6A*-F | AATGGTGGAGACGATGAACC |  |  |
| DJ3-*ERG6A*-R | TTTGCGAGCCTATTAATGACGA |  |  |
| *ERG6A*-Check-F | CAAGGACTCGAGAAGGAGGA | Validation primers for *ERG6A* knockout transformants | |
| *ERG6A*-Check-R | TCAGTTCATGGGCTTGCGT |  |  |
| DJ1-*ERG6C*-5F | TTCGGATGGCAACCTGAAAC | *ERG6C* knockout primers | |
| DJ1-*ERG6C*-5R | TCAATATCATCTTCTGTCGAGGATGAGATGGGGATGGGAT |  |  |
| DJ1-*ERG6C*-3F | AGATGCCGGATCCACTTAACAGGTTAGCAGACAGGCGAAA |  |  |
| DJ1-*ERG6C*-3R | GCTGTACAGGGGTAGAGATG |  |  |
| DJ3-*ERG6C*-F | ACCCTCACAACAAGAATAGGT |  |  |
| DJ3-*ERG6C*-3R | ACATCGACATCACATGAAGC |  |  |
| *ERG6C*-Check-F | ATGTCCAAACAACAGTATCAAA | Validation primers for *ERG6C* knockout transformants | |
| *ERG6C*-Check-R | TTACTCTTCCGCAAACGGC |  |  |
| DJ1-*SDR13*-5F | ACCTCCTCCAATATGACGTG | *SDR13* knockout primers | |
| DJ1-*SDR13*-5R | TCAATATCATCTTCTGTCGATTAATTGCCCTCGGACCCTT |  |  |
| DJ1-*SDR13*-3F | AGATGCCGGATCCACTTAACGTTTTACTGGGAGCTGGCTC |  |  |
| DJ1-*SDR13*-3R | AATGCTAGACAAAGCGTGGA |  |  |
| DJ3-*SDR13*-F | CGCATCATCCAATTGTAGCC |  |  |
| DJ3-*SDR13*-R | ATTTGTTGCACAAAGCGACG |  |  |
| *SDR13*-Check-F | ATGTCCGTAACATGCAGCTT | Validation primers for *SDR13* knockout transformants | |
| *SDR13*-Check-R | CTAAGCATCCACCTTTACAAAGC |  |  |
| DJ1-*CYP51A*-5F | GCGATGGCGTATAAAGATTTT | *CYP51A* knockout primers | |
| DJ1-*CYP51A*-5R | TCAATATCATCTTCTGTCGACATGACAACTGGTAATATGCCAG |  |  |
| DJ1-*CYP51A*-3F | AGATGCCGGATCCACTTAACTTGATCCAACCAAGGCTGGA |  |  |
| DJ1-*CYP51A*-3R | TGTGGGATATGACACCAGCT |  |  |
| DJ3-*CYP51A*-F | CAGTTTTGGTGCTGGAGAGT |  |  |
| DJ3-*CYP51A*-R | CAAAACGGTCGGGATGAAGT |  |  |
| *CYP51A*-Check-F | ATGGCTTTCTTCTTCCCATC | Validation primers for *CYP51A* knockout transformants | |
| *CYP51A*-Check-R | CTACGCAGTCTTCGGGCTG |  |  |
| DJ1-*CYP51B*-5F | GTTCGGACCGGCAGAGCATA | *CYP51B* knockout primers | |
| DJ1-*CYP51B*-5R | TCAATATCATCTTCTGTCGACATCCTTGTGGAAATCTCTCCT |  |  |
| DJ1-*CYP51B*-3F | AGATGCCGGATCCACTTAACAGGGGCAAGAGAAAAACAAAAAG |  |  |
| DJ1-*CYP51B*-3R | GCGAGGAGGTGGCAACAG |  |  |
| DJ3-*CYP51B*-F | CGACATACGTGTACGCGAAT |  |  |
| DJ3-*CYP51B*-R | TCTTCAGCGACACGTGCC |  |  |
| *CYP51B*-Check-F | ATGGGCCTTCTACAGGACAC | Validation primers for *CYP51B* knockout transformants | |
| *CYP51B*-Check-R | AAATCGGCCAGGGTCTCG |  |  |
| DJ1-CYP51A1-Bar-3F | cccgccttcagtttaaactatcTTGATCCAACCAAGGCTGGA | *MoDde1* and *CYP51A* knockout primers | |
| DJ1-CYP51A1-Bar-5R | AACGTATCTTATCGAGATCCTGAACCATGACAACTGGTAATATGCCAGA |  |  |
| qPCR-*MoCYP51A*-F | TTCGACGGTCACGTATGG | Primers for evaluating *MoCYP51A* expression | |
| qPCR-*MoCYP51A*-R | TGCCGATGTAGACGGTTGT |  |  |
| qPCR-*MoCYP51B*-F | TTCGACGGTCACGTATGG | Primers for evaluating *MoCYP51B* expression | |
| qPCR-*MoCYP51B*-R | TGCCGATGTAGACGGTTGT |  |  |
| qPCR-*FgCYP51A*-F | CAAGGAAATCGCCCACAT | Primers for evaluating *FgCYP51A* expression | |
| qPCR-*FgCYP51A*-R | TCGGGAGAAGAAGCAAGGT |  |  |
| qPCR-*FgCYP51B*-F | CTACGGTTACGGCCTTGT | Primers for evaluating *FgCYP51B* expression | |
| qPCR-*FgCYP51B*-R | TTGCGGAACTTGAACTCG |  |  |
| qPCR-*FgCYP51C*-F | ATCGTCCACCCGTTGTAT | Primers for evaluating *FgCYP51BC* expression | |
| qPCR-*FgCYP51C*-R | GCAGTCGCCGTATTTATCT |  |  |
| qPCR-*SsCYP51B*-R | AGGGTCCATACCATAAGTGA | Primers for evaluating *SsCYP51B* expression | |
| qPCR-*SsCYP51B*-F | GCAGCATTCGTCGTTCTA |  |  |
| qPCR-*MfCYP51B*-F | CTCCCGCTATGGCTGAAA | Primers for evaluating *MfCYP51B* expression | |
| qPCR-*MfCYP51B*-R | CCATGTCGAGGTCGTGGT |  |  |
| qPCR-*BcCYP51B*-F | AGCGTTCTTCGGCGTTCA | Primers for evaluating *BcCYP51B* expression | |
| qPCR-*BcCYP51B*-R | CCATGTCGAGGTCGTGGT |  |  |
| qPCR-*MoActin*-F | TTCCTATTTACGAGGGTTTCT | Primers for evaluating *MoActin* expression | |
| qPCR-*MoActin*-R | GATGTCACGGACGATTTCT |  |  |
| qPCR-*FgActin*-F | GATGTCGATGTCCGAAAGG | Primers for evaluating *FgActin* expression | |
| qPCR-*FgActin*-R | GGAAGAAGGAGCAAGAGCA |  |  |
| qPCR-*SsActin*-F | CTTCCATACCAGCGCAGAG | Primers for evaluating *SsActin* expression | |
| qPCR-*SsActin*-R | CACCCGACCAGTCCTTTT |  |  |
| qPCR-*MfActin*-F | CCCCAATCAACCCAAAGT | Primers for evaluating *MfActin* expression | |
| qPCR-*MfActin*-R | CTCCGTCACCAGAATCGA |  |  |
| qPCR-*BcActin*-F | TGGCTGGTCGTGATTTGA | Primers for evaluating *BcActin* expression | |
| qPCR-*BcActin*-R | TGGCGGTTTGGATTTCTT |  |  |
| qPCR-*MoDde1*-F | CCCAGAACCAGTTCGTCAG | Primers for evaluating *MoDde1* expression | |
| qPCR-*MoDde1*-R | GCCAGCCAGCTTCACATC |  |  |
| qRT-*MoSR*-F | AGAAATAGTCCGAGCATCCC | Primers for evaluating *MoSR* expression | |
| qRT-*MoSR*-R | GCAACGACCGACTCATCC |  |  |
| pHIS2-*MoCYP51A*-F | GACTCACTATAGGGCGAATTCAGCCCTCGCATCTCAA | Primers for *MoCYP51A* promoter in yeast one hybrid | |
| pHIS2-*MoCYP51A*-R | ATAATGCCAGGAATTACTAGTATATGGCCCCAAACAGAAC |  |  |
| pHIS2-*MoCYP51B*-F | GACTCACTATAGGGCGAATTCCAAAGCTAGGCTTGGGCG | Primers for *MoCYP51B* promoter in yeast one hybrid | |
| pHIS2-*MoCYP51B*-R | ATAATGCCAGGAATTACTAGTGCGAATTCGGTTTCTCTAAT |  |  |
| pHIS2-*MoDde1*-F | GACTCACTATAGGGCGAATTCTTTGCCTTGCAGCTGCTAC | Primers for *MoDde1* promoter in yeast one hybrid | |
| pHIS2-*MoDde1*-R | ATAATGCCAGGAATTACTAGTTGCAAGTACGGCCACGGAG |  |  |
| AD-*MoSR*-F | CCATGGAGGCCAGTGAATTCATGCCGCCGCGACGCTCA | Primers for *MoSR* in yeast one hybrid | |
| AD-*MoSR*-R | GCTCGAGCTCGATGGATCCCTCAAGAGCTGCTTCGGGCAG |  |  |
| 62K-*MoSR*-F | CCCCCGGGCTGCAGGAATTCATGCCGCCGCGACGCTCACA | Primers for *MoSR* in dual-luciferase reporter systems | |
| 62K-*MoSR*-R | GTCGACGGTATCGATAAGCTTAGAGCTGCTTCGGGCAGGC |  |  |
| 800-*MoDde1*-F | CGAGGTCGACGGTATCGATTTTGCCTTGCAGCTGCTACGT | Primers for *MoDde1* promote in dual-luciferase reporter systems | |
| 800-*MoDde1*-R | TCTAGAACTAGTGGATCCTGCAAGTACGGCCACGGAGTAT |  |  |
| PTNHG-*MoDde1*-F | CACTGCAGCCCGGGGGATCCATGTCGACGACGCAACTCGC | Primers for *MoDde1-GFP* amplification in overexpression transformants | |
| PTNHG-*MoDde1*-R | CCAGCACCAGCACCTCTAGAAGCACGCTCCTTGAAAAGAA |  |  |
| PTNHF-*MoDde1*-F | CACTGCAGCCCGGGGGATCCATGTCGACGACGCAACTCGC | Primers for *MoDde1-Flag* amplification in overexpression transformants | |
| PTNHF-*MoDde1*-R | TCATGGTCTTTGTAGTCCATAGCACGCTCCTTGAAAAGAA |  |  |
| PTNG-*MoDde1*-F | TCGAGGTCGACGGTATCGATCTCGAACAAGCAGAGCGAAA | Primers for *MoDde1* amplification in complementation transformants | |
| PTNG-*MoDde1*-R | CCTCTAGAACTAGTGGATCCAGCACGCTCCTTGAAAAGAA |  |  |
| pYES2-*MoCYP51A*-F | CTATAGGGAATATTAAGCTTATGGCTTTCTTCTTCCCATC | Primers for *MoCYP51A* amplification in yeast BY4741 | |
| pYES2-*MoCYP51A*-R | CCGTTACTAGTGGATCCTTACGCAGTCTTCGGGCTGCGTCG |  |  |
| pYES2-*MoDde1*-F | CTATAGGGAATATTAAGCTTATGTCGACGACGCAACTCGC | Primers for *MoDde1* amplification in yeast BY4741 | |
| pYES2-*MoDde1*-R | CCGTTACTAGTGGATCCTTAAGCACGCTCCTTGAAAAGAA |  |  |
| HIGS-*MoDde1*-F1 | GGTACCGCACGACGGCGCCTTCACCG | Primers for *MoDde1* amplification in HIGS | |
| HIGS-*MoDde1*-R1 | GGATCCTCGTAGTTGAGGACCAGCCT |  |  |
| HIGS-*MoDde1*-F2 | ACTAGTGCACGACGGCGCCTTCACCG |  |  |
| HIGS-*MoDde1*-R2 | GAGCTCTCGTAGTTGAGGACCAGCCT |  |  |

**Figure S1.** Functional validation of *MoSR* in *M. oryzae*. (A) Targeted knockout strategy for *MoSR* with schematic representation (left) and validation of Δ*MoSR* transformants by PCR using specific primers (right). (B) Complementation vector design (top) and transcript restoration verification in complemented transformants Δ*MoSR*-C through RT-PCR (bottom).

**Figure S2.**  Fitness evaluation of *MoSR*-related transformants in *M. oryzae*. (A) Comparative growth rates of *MoSR*-related transformants on complete medium (CM), potato dextrose agar (PDA), and oat tomato agar (OTA). (B-E) Sporulation capacity, conidial germination rates, appressorium formation, and pathogenicity assays of *MoSR*-related transformants. (F) Mycelial inhibition rate of *MoSR* related transformants to different stress agents. Transformants were inoculated on PDA or PDA amended with 0.7 M NaCl, 0.8 M KCl, 1.2 M sorbitol (SOR), 0.025% SDS (w/v), 1200 μg/mL Congo red (CR), 1200 μg/mL calcofluor white (CFW) and 6 mM H_2_O_2_ and then incubated at 27℃ for 5 days. Data presented are the mean ± SE (n = 9). Bars followed by the same letter are not significantly different according to a LSD test at *P* = 0.01.

**Figure S3.** Molecular validation of *MoDde1* gene knockout, complementation and overexpression. (A) Knockout strategy (top) and verification by primer-specific PCR (bottom). (B) Complementation vector design (top) and RT-PCR confirmation of *MoDde1* expression in Δ*MoDde1*-C transformant (bottom). (C) Overexpression vector schematic (Top) and verification of overexpression transformants OE*MoDde1* by Western blot using anti-GFP antibodies (bottom).

**Figure S4.** Sensitivity of knockout transformants of MoSR-regulated genes in *M. oryzae*. (A) Fungicide sensitivity assays of *MoDde1* related transformants to other DMI fungicides (0.025 μg/mL prochloraz (Proc), 0.5 μg/mL ketoconazole (Ket), 0.6 μg/mL propiconazole (Prop), 0.8 μg/mL econazole (Eco), 0.2 μg/mL diniconazole (Din, 1 μg/mL triadimefon (Tri)). (B) Data analysis of the sensitivity of *MoDde1* related transformants to other DMI fungicides. (C) Mycelial growth inhibition assays of *MoDde1*-related transformants exposed to diverse fungicides, i.e., 4 μg/mL fludioxonil (Flu), 20 μg/mL iprodione (Ipr), 5 μg/mL isoprothiolane (IPT), 0.3 μg/mL carbendazim (Car), 1 μg/mL azoxystrobin (Azo), and 30 μg/mL boscalid (Bos). (D) Data analysis of the sensitivity of *MoDde1* related transformants to six fungicides. Wild-type H08-1a and transformants were cultured on PDA plates with/without fungicides at 27°C for 5 days. Data represent mean ± SD. Bars followed by the same letter are not significantly different according to a LSD test at *P* = 0.01.

**Figure S5.** Fitness of *MoDde1* knockout and complemented transformants. (A) Colony growth of wild-type H08-1a, Δ*MoDde1*, and Δ*MoDde1*-C strains on complete medium (CM), minimal medium (MM), potato dextrose agar (PDA), and oat tomato agar (OTA). (B-D) Reproductive and developmental fitness parameters of *MoDde1* related transformants, i.e., sporulation (B), conidial germination (C), and appressorium formation (D). (E) Pathogenicity assay on *Oryza sativa* cv. ZH11 leaves following 10-day post-inoculation with *MoDde1* related transformants. Data presented are the mean ± SE (n = 9). (F, G) Stress tolerance under osmotic stressors (0.7 M NaCl, 0.8 M KCl, 1.2 M sorbitol (SOR)), cell wall/membrane perturbants (0.025% SDS, 1200 μg/mL Congo red (CR), 1200 μg/mL calcofluor white (CFW)), and oxidative stress (6 mM H₂O₂), colony growth phenotypes and statistical analysis were indicated in F and G, respectively. All experiments were conducted at 27°C for 5 days unless specified. Data presented are the mean ± SD (n = 6). Bars followed by the same letter are not significantly different according to a LSD test at *P* = 0.01.

**Figure S6.** Phylogenetic analysis and functional validation of MoDde1 homologs. (A) Phylogenetic tree of MoDde1 homologs across fungal taxa, based on NCBI-derived amino acid sequences. Homologs are restricted to Sordariomycetes (e.g., *Fusarium* spp.), with no detectable orthologs in Leotiomycetes. Amino acid sequences were aligned by ClustalW, the tree was constructed by using Maximum-Likelihood algorithm in MEGA 7.0). (B) Overexpression transformant OE*FgDde1* increased resistance to Teb. (C) Statistical data of mycelial inhibition in overexpression transformant OE*FgDde1* to Teb. *pTrpC-FgDde1* indicates three independent over-expression mutants (*pTrpC-*FgDde1‑1, *pTrpC-*FgDde1‑2, *pTrpC-*FgDde1‑3). Data presented are the mean ± SD (n = 6). (D) Expression level of *FgDde1* in OE*FgDde1* transformant detected by RT-qPCR. Data were normalized to *FgActin* and presented as mean ± SD (n=3). (E) The *MoDde1* heterologous expression transformant *pEF1-MoDde1* in *Botrytis cinerea* significantly increased resistance to Teb. (F) Statistical data of mycelial inhibition in *MoDde1* heterologous expression transformant *pEF1-MoDde1* to Teb. (G) Protein level of *MoDde1*in *pEF1-MoDde1* transformant detected by Western blot. (H) FgDde1 is not involved in regulating fungal sensitivity to Tri in *F. graminearum*. Data presented are the mean ± SE (n = 9). (I) Molecular docking simulations verified the stable binding interaction between FgCYP637A1 and Tri. Statistical significance was analyzed using the t-test. Two asterisks (**) indicate *P* < 0.01, and “ns” denotes no significant difference.

**Figure S7.** DARTS experiments confirmed the binding interaction between MoDde1 and two triazole fungicides, tebuconazole and triadimefon. (A) Analyze the cleavage efficiency of MoDde1 under different proteinase K concentrations. (B) The interactions between MoDde1 and tebuconazole/triadimefon were verified with 1:1000 diluted proteinase K. Phenylmethylsulfonyl fluoride (PMSF) is a widely used protease inhibitor that can suppress protease activity.

**Figure S8.** Structure and activity analysis of small molecule inhibitors of MoDde1 and selection strategy of silencing fragments of MoDde1. (A) Chemical structures of five small molecule inhibitors. (B) Activity analysis of five small molecule inhibitors. The wild type H08-1a, and Δ*MoDde1* transformant were inoculated on PDA or PDA plates amended with 0.3 µg/mL Teb, 1 µg/mL inhibitors , and mixed. (C) Statistical data of mycelial inhibition in H08-1a, and Δ*MoDde1* to Teb, inhibitors (-) or mixed (+). Data presented are the mean ± SD (n = 3). (D) The small-molecule inhibitor D1 exhibits specific inhibitory activity toward MoDde1, with no observable suppression of MoCYP51A or FgCYP637A1 activity. (E) The combination of D1 and Teb did not improve the Teb sensitivity in *F. graminearum*. Data presented are the mean ± SEM (n = 9). Statistical analysis was performed using the t-test, and “ns” indicates no significant difference. (F) Spray inoculation experiments demonstrated that combined application of Teb and D1 significantly enhanced the control efficacy of Teb against rice blast. Data presented are the mean ± SD (n > 50). (G) Construction strategy of HIGS-Modde1 rice silencing fragment. HIGS vector is DS1301. (H) Spray inoculation experiments verified that rice lines with silenced *MoDde1* markedly improved the control efficacy of Teb against rice blast. Data presented are the mean ± SD (n > 35). Bars followed by the same letter are not significantly different according to a LSD test at *P* = 0.01.
